# Integrating Community-Based Interventions to Reverse the Convergent TB/HIV Epidemics in Rural South Africa

**DOI:** 10.1371/journal.pone.0126267

**Published:** 2015-05-04

**Authors:** Jennifer A. Gilbert, Elisa F. Long, Ralph P. Brooks, Gerald H. Friedland, Anthony P. Moll, Jeffrey P. Townsend, Alison P. Galvani, Sheela V. Shenoi

**Affiliations:** 1 Department of Epidemiology of Microbial Diseases, Yale School of Public Health, New Haven, CT, United States of America; 2 Center for Infectious Disease Modeling and Analysis, Yale School of Public Health, New Haven, CT, United States of America; 3 Anderson School of Management, University of California Los Angeles, Los Angeles, CA, United States of America; 4 Department of Medicine, Section of Infectious Diseases, AIDS Program, Yale University School of Medicine, New Haven, CT, United States of America; 5 Church of Scotland Hospital, Tugela Ferry, KwaZulu-Natal, South Africa; 6 Department of Biostatistics, Yale University, New Haven, CT, United States of America; 7 Department of Ecology and Evolutionary Biology, Yale University, New Haven, CT, United States of America; 8 Program in Computational Biology and Informatics, Yale University, New Haven, CT, United States of America; University of Delhi, INDIA

## Abstract

The WHO recommends integrating interventions to address the devastating TB/HIV co-epidemics in South Africa, yet integration has been poorly implemented and TB/HIV control efforts need strengthening. Identifying infected individuals is particularly difficult in rural settings. We used mathematical modeling to predict the impact of community-based, integrated TB/HIV case finding and additional control strategies on South Africa’s TB/HIV epidemics. We developed a model incorporating TB and HIV transmission to evaluate the effectiveness of integrating TB and HIV interventions in rural South Africa over 10 years. We modeled the impact of a novel screening program that integrates case finding for TB and HIV in the community, comparing it to status quo and recommended TB/HIV control strategies, including GeneXpert, MDR-TB treatment decentralization, improved first-line TB treatment cure rate, isoniazid preventive therapy, and expanded ART. Combining recommended interventions averted 27% of expected TB cases (95% CI 18–40%) 18% HIV (95% CI 13–24%), 60% MDR-TB (95% CI 34–83%), 69% XDR-TB (95% CI 34–90%), and 16% TB/HIV deaths (95% CI 12–29). Supplementing these interventions with annual community-based TB/HIV case finding averted a further 17% of TB cases (44% total; 95% CI 31–56%), 5% HIV (23% total; 95% CI 17–29%), 8% MDR-TB (68% total; 95% CI 40–88%), 4% XDR-TB (73% total; 95% CI 38–91%), and 8% TB/HIV deaths (24% total; 95% CI 16–39%). In addition to increasing screening frequency, we found that improving TB symptom questionnaire sensitivity, second-line TB treatment delays, default before initiating TB treatment or ART, and second-line TB drug efficacy were significantly associated with even greater reductions in TB and HIV cases. TB/HIV epidemics in South Africa were most effectively curtailed by simultaneously implementing interventions that integrated community-based TB/HIV control strategies and targeted drug-resistant TB. Strengthening existing TB and HIV treatment programs is needed to further reduce disease incidence.

## Introduction

South Africa has the highest burden of TB/HIV co-infection worldwide [1]. Complicating the TB/HIV co-epidemics is the spread of drug resistant TB, with 2–7% of TB cases resistant to the first-line drugs isoniazid and rifampicin (*i*.*e*., multidrug-resistant or MDR-TB), 10% of which are also resistant to any fluoroquinolone and at least one injectable second-line drug (*i*.*e*., extensively drug-resistant or XDR-TB) [1,2]. Community transmission of drug resistant TB continues to increase in South Africa [2] and globally [1], galvanizing the design of innovative approaches to strengthen TB treatment programs and hospital infection control measures [3–5].

The WHO recommends the integration of TB and HIV control, but implementation within South Africa has fallen below the recommendations [5,6]. Improving the first-line TB treatment cure rate, isoniazid preventive therapy (IPT) for HIV infected individuals, and expanding antiretroviral therapy (ART) coverage to reduce TB incidence in countries with high HIV prevalence [1,6,7] are goals of the WHO. National South African policies also advocate improving ART initiation, default, and coverage [8], as well as decentralizing MDR-TB treatment to facilitate treatment initiation and reduce treatment default [9]. The PCR-based GeneXpert assay is a novel diagnostic technology [10] that has been assessed to be effective at reducing the delay in diagnosis and treatment of MDR-TB [11], but has yet to be fully examined in the context of or compared to other TB interventions.

Current TB and HIV control in South Africa generally relies on passive case finding, where symptomatic patients self-present to healthcare facilities [12]. In contrast, intensified case finding seeks out cases by actively screening in the community or other settings. Intensified TB case finding has been predicted to reduce TB in high incidence settings [13–15], while expanded HIV screening and ART within the community have been shown to reduce HIV and TB incidence [16,17]. A recent clinical trial examined the impact of combining TB and HIV screening in the community and households, but coverage of the screening was only a few percent and did not detect a statistically significant impact on TB incidence [18]. Additionally, this clinical trial did not consider the effects of the intervention on HIV incidence, nor did it target TB drug resistance. A novel intensified case finding strategy that integrates TB and HIV screening while also targeting drug resistant TB was recently implemented in rural KwaZulu-Natal, South Africa. Specifically, community-based integrated TB/HIV intensified case finding (CICF) complements recommended prevention and treatment interventions, as well as passive case finding strategies, by combining TB and HIV screening in the community and linking diagnosed individuals to the appropriate medical care and treatment. The population level impact of CICF has yet to be investigated.

Clinical and field studies of TB and HIV interventions have focused on the health outcomes of individual patients [10,19–21]. Because interventions can have a public health impact beyond the treated patient by reducing transmission to others, mathematical modeling that incorporates transmission dynamics is useful to estimate the population level impacts of an intervention. Modeling is particularly valuable for predicting dynamical non-linearities arising from combining interventions. Although the population level effects of improved first-line TB treatment cure rate, expanded ART coverage, IPT, and GeneXpert on the TB epidemic have been investigated individually [11,16,22–25], the impact of combining these interventions—along with MDR-TB treatment decentralization and CICF—on the TB/HIV co-epidemics remains to be evaluated [26].

To determine the comparative effectiveness of integrated TB and HIV interventions on the convergent TB/HIV epidemics in South Africa, we developed a model that incorporates the dynamics of both TB and HIV. Comparing interventions implemented separately and in combination, we predict reductions in the number of TB and HIV cases, MDR and XDR-TB cases, and TB and HIV mortality resulting from the integration of CICF with the following recommended TB and/or HIV interventions relative to status quo disease control: GeneXpert, MDR-TB treatment decentralization, improved first-line TB treatment cure rate, IPT, and expanded ART. We also examined the interactions between CICF and recommended TB and HIV interventions to determine the contribution of this new, integrated screening strategy to the reduction in TB and HIV incidence and mortality in the context of current control strategy recommendations. Finally, we used sensitivity analysis to determine which TB and HIV control parameters most greatly influenced the percentages of TB and HIV cases and deaths averted.

## Materials and Methods

### Overview

**Fig 1:**
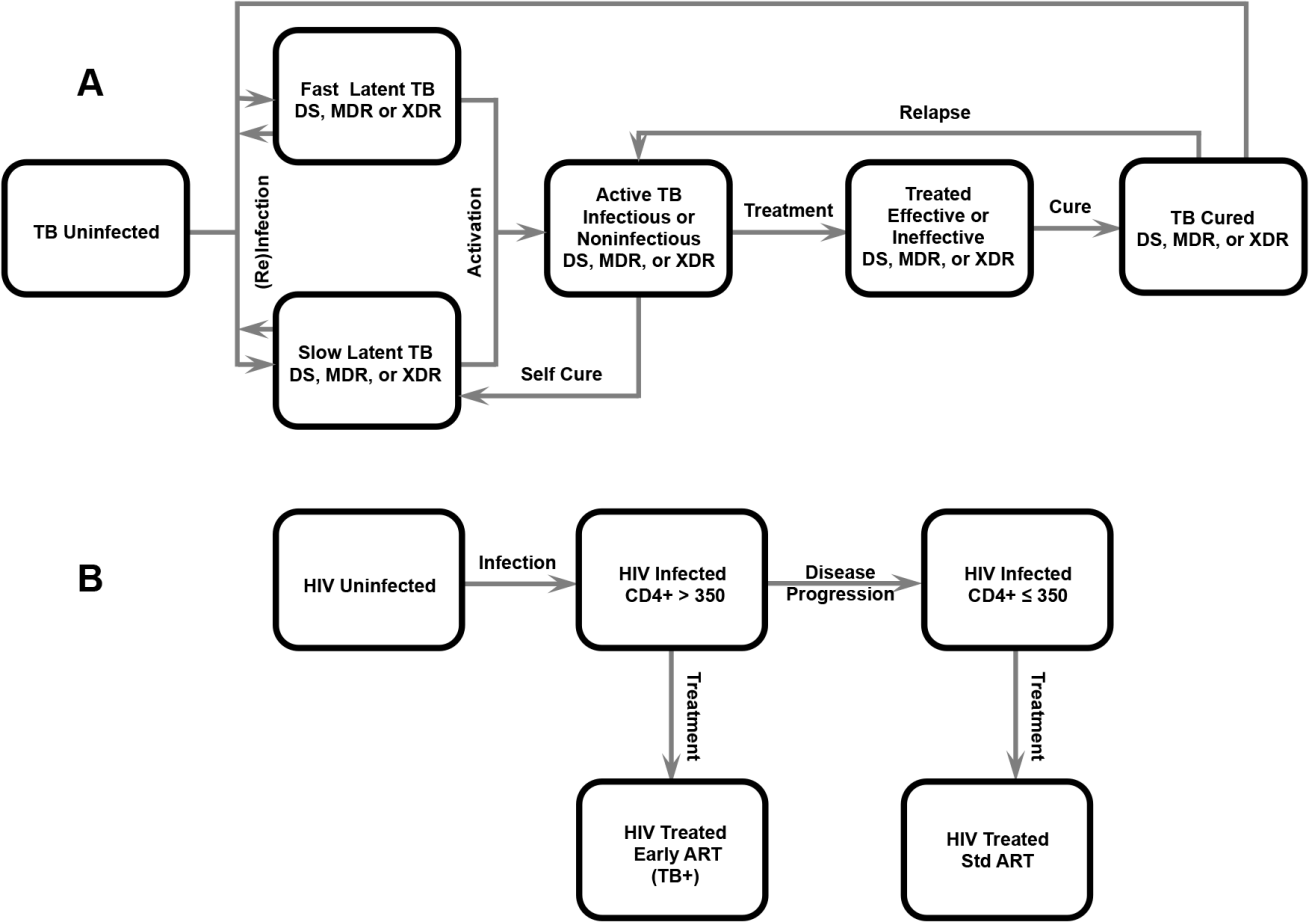
Model Diagrams. Model diagrams of (A) TB transmission dynamics and (B) HIV transmission dynamics. Although depicted separately, modeled individuals had a status for each disease. Drug resistance (*i*.*e*., drug susceptible [DS], multidrug-resistant [MDR], and extensively drug-resistant [XDR] TB) was also included in the model. Model parameters and equations are detailed in S1 Text.

We developed a dynamic model of community-based TB and HIV to examine the potential impact of integrated TB and HIV interventions on the co-epidemics among adults aged 15–64 in a rural area of South Africa (Fig 1), calibrated to epidemiological data for both diseases and validated against epidemiological and demographic data (see S1 Text). We modeled a rural community of 137,000 people, representing the adult population of the Msinga subdistrict of KwaZulu-Natal [27]. The compartmental model structure is detailed below and model equations and parameter values are presented in S1 Text. We first estimated the status quo 10 year cumulative incidence of drug susceptible TB, MDR-TB, XDR-TB, and HIV, as well as disease-associated deaths, and compared with projections when the following interventions were implemented individually and in combination: GeneXpert screening test for MDR-TB, MDR-TB treatment decentralization, improved first-line TB treatment cure rate, IPT, ART expansion, and CICF.

### Model

#### TB Transmission Dynamics

We modeled drug susceptible, MDR, and XDR-TB, including acquired and amplified resistance during therapy, transmission of susceptible and resistant strains, self-cure, strain fitness, exogenous re-infection, and mortality [3], parameterized from clinical and epidemiological data (S1 Table). Transitions in the model occur from susceptibility to infection with tuberculosis and progression from active disease to treatment and/or recovery. Consistent with other models of TB infection [28], susceptible individuals become infected with TB and develop a latent infection of either short or long duration. The rates of drug susceptible, MDR, and XDR-TB transmission are proportional to the prevalence of drug susceptible, MDR, and XDR-TB, respectively, in the population [27,29]. Latently-infected individuals progress to active infectious or non-infectious TB disease. Non-infectious individuals may become infectious over time [24,30], and a small proportion of smear-negative patients are classified as infectious, consistent with clinical data [31]. Drug efficacy, adherence, and default collectively determine the effectiveness of active TB treatment [1]. Individuals who are effectively treated recover, while ineffectively treated individuals remain infectious and may experience acquired or amplified resistance [32]. A small number of active TB patients or ineffectively treated individuals may also self-cure and return to a latently-infected state [25]. Some patients who recover via treatment may relapse to active disease [25]. Latently-infected and recovered individuals can be exogenously re-infected with any type of TB (*i*.*e*., drug susceptible, MDR, or XDR), subject to partial immunity and the time-dependent risk of infection with that type [33].

#### HIV Transmission Dynamics

We modeled early and late stages of HIV disease, prevalence-dependent behavior change, as well as ART initiation, loss to follow-up, default, and mortality. Individuals uninfected with HIV, and therefore susceptible, can become infected with HIV at a rate proportional to the prevalence of HIV in the population [27,29]. In line with clinical recommendations in 2014 and earlier, HIV infected individuals become eligible for ART when their disease progresses and CD4+ cell count drops below 350 cells per milliliter [34], a calibrated proportion of whom begin ART [27,29]. As is also recommended, HIV infected individuals who also have active TB may begin ART irrespective of CD4+ cell count [34].

#### TB/HIV Coinfection

We account for both the impact of HIV and ART on TB pathogenesis, as well for TB on HIV pathogenesis, such that an individual’s rates of disease progression are dependent on the status of the other disease [35]. We additionally account for these disease interactions by fitting background and disease-specific mortality rates—which depend on TB and HIV disease and treatment status—to longitudinal TB and HIV incidence and prevalence data [27,29]. In general, HIV infection increases the probability of progressive primary disease in those infected with TB, the rate at which latent TB progresses to active disease, and mortality from active TB [36]. HIV infection also reduces the efficacy of TB treatment, and the partial immunity to reinfection or superinfection [36]. Similarly, TB lowers CD4 cell counts, accelerates HIV progression to AIDS, and increases mortality [35,37].

#### Model Calibration and Validation

Data for the construction, calibration, and validation of the model were obtained from the WHO [29] and the Actuarial Society of South Africa (ASSA) [27], as well as from epidemiological studies in rural South Africa (S1 Fig and S1 and S2 Tables). Background and disease-specific mortality, TB and HIV transmission, and treatment rates were fit to longitudinal data on TB incidence, prevalence, and case detection rate from the WHO between 2001 and 2011, as well as HIV prevalence from the Actuarial Society of South Africa [27,29]. These observations were additionally validated using longitudinal TB and HIV incidence and prevalence data, MDR and XDR-TB data, reported TB case detection rates, and ART coverage estimates [2,27,29,38]. Because there were not sufficient data available to distinguish between status quo TB treatment rates for HIV infected and uninfected individuals, or between status quo HIV treatment rates for individuals with and without active TB disease, we assumed that status quo treatment rates for TB disease and HIV infection were independent of coinfection status.

#### Uncertainty and Sensitivity Analyses

To account for how empirical uncertainties in parameter values affect the robustness of model outcomes, we performed uncertainty and sensitivity analyses. We used Latin hypercube sampling from parameter distributions, as described in S1 Text, to generate uncertainty distributions for all model outcomes, and 95% confidence intervals were calculated for model predictions from these distributions. A global sensitivity analysis using partial rank correlation coefficients (PRCCs) was performed to determine which parameters had the greatest influence on model predictions.

### Intervention Strategies

We compared the impact of different strategies, both individually and in combination, on the disease incidence and mortality arising from drug susceptible and drug resistant TB and HIV, as outlined below.

#### Status Quo TB and HIV Detection and Treatment

We compared the effectiveness of potential interventions against a baseline of status quo detection and treatment that is currently implemented in rural South Africa. Specifically, individuals with TB and/or HIV symptoms must present to a clinic or hospital for symptom detection (*e*.*g*., cough lasting longer than two weeks, fever, night sweats, and/or weight loss), sputum smear, and/or chest x-ray. These methods have limitations in both sensitivity and specificity for the diagnosis of TB. In particular, the traditional diagnostic protocols cannot distinguish between drug susceptible and drug resistant TB. Consequently, upon initial diagnosis, TB patients are enrolled in first-line therapy, which involves the administration of four oral antibiotics for up to six months. TB patients who remain symptomatic and/or smear positive after two to three months of TB first-line therapy are then suspected of having drug resistant TB and have culture and subsequent drug susceptibility testing (DST) performed [39]. Culture is the gold standard for diagnosis of active pulmonary TB disease, but culture combined with DST can require over three months to yield results [40,41]. Patients with confirmed MDR or XDR-TB are then referred to a hospital for second-line therapy, which involves at least 24 months of oral and injectable antibiotics [9,39]. This long duration of hospitalized treatment leads to high rates both of default before treatment initiation (50%) [9] and during treatment (14–29%) [2,42,43]. Given that GeneXpert is generally not yet available in rural South Africa, we evaluate the effectiveness of GeneXpert to screen patients for TB and rifampicin resistance as a supplemental intervention to the baseline status quo, and as detailed in the GeneXpert section below.

Patients are typically diagnosed with HIV by an antibody test [44]. Patients are then recommended to receive a CD4+ cell count to determine their eligibility for ART, but many patients do not follow up for the required phlebotomy [45]. Eligible patients are referred to their local ART clinic to initiate ART, but only around half start treatment [46,47], and about 25% of those patients who start treatment default within three years [48].

#### Community-Based Integrated Intensified Case Finding (CICF)

Community-based integrated intensified case finding (CICF) integrates screening and linkage to care for both TB and HIV. CICF is administered to any individuals within the community who are interested in voluntary TB or HIV testing. In its current implementation in Msinga, individuals are screened at congregate settings for TB symptoms using a symptom questionnaire (sensitivity of 69–79%) [49,50]. In contrast to national treatment guidelines, which recommend sputum culture and DST only after first-line treatment fails [51], CICF teams collect sputum from symptomatic individuals for smear, culture, and DST (sensitivity of 68–100%) [41]. Individuals with negative sputum smear and negative sputum culture do not undergo additional evaluation. Individuals diagnosed by sputum smear and/or culture are referred for the appropriate first or second-line TB treatment. Individuals also receive a rapid HIV antibody test (sensitivity of 100%) [44]. Those who test positive have a blood sample taken at the screening site for a CD4+ cell count to determine their ART eligibility status, and if eligible, are referred to their local ART clinic for treatment initiation. We assume individuals with a CD4+ cell count ≤ 350 cells per milliliter to be eligible for ART, as has been recommended by South African ART guidelines prior to 2015 [45]. We also considered the possibility of expanding ART eligibility to all individuals with a CD4+ cell count ≤ 500 cells per milliliter [52], optimistically assuming that the ART coverage of individuals with a CD4+ cell count between 350 and 500 would be equal to the ART coverage of individuals with a CD4+ cell count ≤ 350.

One CICF team screens up to 25 individuals per day, covering approximately 6% of adult Msinga population annually [27]. We evaluated the effectiveness of increasing the frequency of CICF screening individuals in the community from on average once every five years to five times annually, corresponding to between two and 85 teams, respectively. For comparisons with other interventions, we focused on an annual CICF screening frequency of on average once every year, which is South Africa’s goal for TB screening and could be accomplished by a scaling up to 17 teams [12].

#### GeneXpert

GeneXpert (Xpert MTB/RIF) is a PCR-based technology that rapidly detects TB with a higher sensitivity than sputum smear (98.3% versus 83.3% for infectious TB, 76.9% versus 0% for noninfectious TB) [3,31,53,54]. GeneXpert can also detect resistance to the first-line TB drug rifampicin (sensitivity of 94.4%) [54] within hours [10]. By contrast, sputum culture and DST require several months [55,56], but GeneXpert has a lower sensitivity, particularly in HIV infected individuals [10].

Although GeneXpert has the potential to replace DST, current plans only call for it to replace sputum smear for baseline TB diagnosis and distinguish between drug susceptible and MDR-TB for initial drug sensitivity screening [57]. We considered supplementing DST with GeneXpert for MDR-TB diagnosis, while continuing to rely on DST for diagnosis of XDR-TB. Although GeneXpert reduces the time to diagnosis of MDR-TB from months to days, initiation of second-line therapy delay due to limited hospital bed availability and loss to follow-up remain [54]. Because empirical treatment of smear negative or smear unknown symptomatic patients is common, the rate of drug susceptible TB diagnosis has been found to be unaffected by GeneXpert relative to baseline [58,59].

While another PCR-based technology called line probe assay (LPA) is also available to diagnose rifampicin resistance, unlike GeneXpert, it is not well suited for decentralized or rural settings in South Africa. LPA can only be used on smear positive patients, and many of South Africa’s TB patients are smear negative due to the low sensitivity of sputum smear microscopy, particularly in HIV positive individuals [12]. LPA can also only be performed in specially ventilated laboratories located in urban areas with results taking seven days to receive, whereas GeneXpert can be used at decentralized locations or for point-of-care diagnosis within 48 hours [60]. Furthermore, South African national guidelines only recommend the use of LPA if GeneXpert is inconclusive on rifampicin resistance [12], highlighting the limited role for LPA in the diagnostic algorithm. Because GeneXpert is superior to LPA in rural South African settings, we did not consider LPA as a potential intervention in our modeled population. We would expect that if implemented, LPA would reduce MDR and XDR-TB incidence, but to a much lesser degree than with GeneXpert.

#### MDR-TB Treatment Decentralization

To reduce delays to second-line treatment initiation [9] and treatment default (14%–29%) [2,42,43], new guidelines recommend shorter hospitalization and home-based MDR-TB management where patients are visited by a nurse for the administration of injectable drugs and supervision of oral medications [9]. In addition, decentralized second-line therapy for MDR-TB frees up beds in TB hospitals for XDR-TB patients [9]. Preliminary analysis in KwaZulu-Natal suggests that decentralized care both reduces delays between diagnosis of MDR and XDR-TB and treatment initiation, as well as reduces default from MDR-TB treatment down to the levels for first-line treatment [21]. Specifically, decentralized care reduced the delay to second-line treatment initiation for MDR-TB to a matter of days following diagnosis and reduced treatment default to 7% [19,21].

#### Improved First-line TB Therapy Cure Rate

The estimated TB cure rate with first-line therapy is between 58% and 73%, depending on a patient’s HIV and ART status [3,30,42,55,61–63]. We modeled the impact of increasing the cure rate to the WHO goal of 85%, which could be achieved by improving treatment adherence [1].

#### Isoniazid Preventive Therapy (IPT)

The WHO and South African Department of Health recommends 12 months for TST negative individuals and 36 months for TST positive individuals [7,45,64] and to treat latent infection and prevent progression to active TB [6], typically implemented in conjunction with ART initiation [5,20]. We modeled this IPT strategy, assuming individuals treated with isoniazid cannot be infected with nor reactivate latent infection of drug susceptible TB for the duration of the IPT [7]. We additionally assumed that only patients in care and on ART received IPT, as is currently implemented in South Africa [5]. Upon IPT completion, individuals gradually return to baseline relative risk of TB infection and reactivation [64]. Additionally, IPT does not prevent the reactivation or transmission of drug resistant TB [7].

#### Expansion of ART Coverage

Currently, only about 62% of eligible patients start ART [46], and only 75% of those patients remain on ART after three years [48], in contrast to the highly optimistic parameterization of prior modeling studies investigating the impact of ART on TB in South Africa (*e*.*g*., [17]). We evaluated expanding ART coverage over four years to reflect the goals of the South African Department of Health to achieve 80% coverage and 70% five-year retention by 2016 [8], corresponding to expanding the baseline coverage of 39% (for all patients with CD4+ count ≤ 350) by 24% annually [48]. While we assume that individuals with a CD4+ cell count ≤ 350 cells per milliliter are eligible for ART, as has been recommended by South African ART guidelines prior to 2015 [45], we also considered the possibility of expanding ART eligibility to all individuals a CD4+ cell count ≤ 500 cells per milliliter [52]. Because it is not yet known what the expected ART coverage of individuals with a CD4+ cell count between 350 and 500 would be, we varied the ART coverage of these newly eligible individuals between 0% and 100% of the ART coverage of individuals with a CD4+ cell count ≤ 350.

As ART resistance is estimated to be below 5% in South Africa [65], it was not included in our status quo scenario. Nonetheless, we considered the possibility that ART resistance, and hence treatment efficacy, could become a clinical concern in the future by decreasing ART efficacy from 100% to 50% in adherent patients on model outcomes. We took into account that decreased ART efficacy would lead to higher viral loads, increased infectiousness, and elevated mortality for treated HIV infected individuals.

## Results

### Status Quo

To determine the percentage of cases averted by integrated TB/HIV interventions (*i*.*e*., the percentage reduction in cumulative disease incidence), we first projected the TB and HIV cases and deaths that would occur over 10 years in the modeled population of adults aged 15 to 64 without the addition of interventions to the status quo. For the status quo scenario, we estimated a baseline 10-year cumulative TB incidence of 29,630 in Msinga, including cases with and without HIV coinfection: 27,370 for drug susceptible TB, 1,706 for MDR-TB, 556 for XDR-TB, 69,743 for HIV, and 11,292 TB and/or HIV deaths over 10 years. These correspond to an estimated annual TB incidence of 1,147 per 100,000 population, TB prevalence of 1,052 per 100,000 population, and HIV prevalence of 12% (in the adult population) at the end of the 10 year period.

### CICF

**Fig 2:**
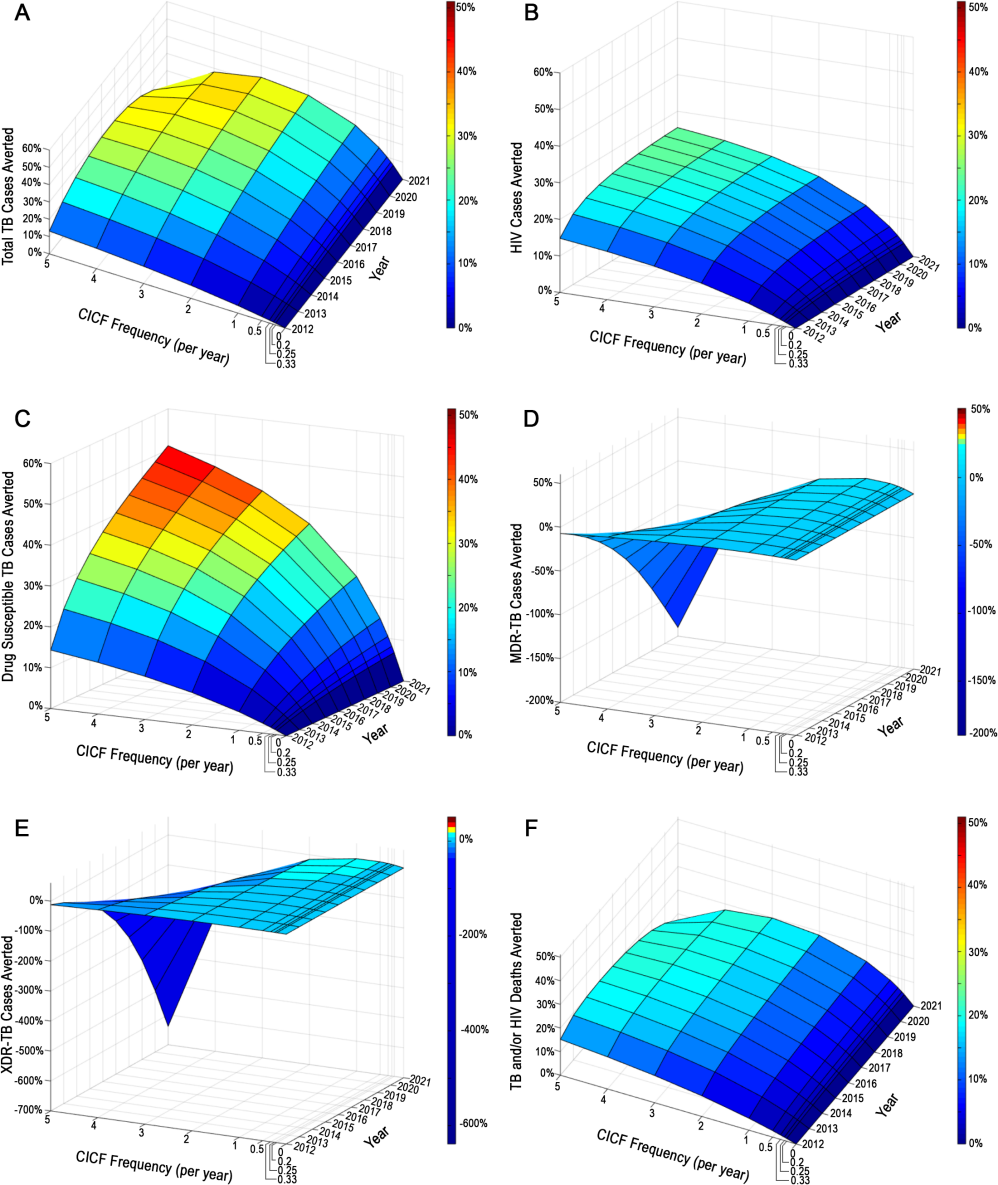
CICF Impact. The impact of CICF over a 10 year period on (A) total TB cases averted, (B) HIV infections averted, (C) drug susceptible TB cases averted, (D) MDR-TB cases averted, (E) XDR-TB cases averted, and (F) TB and HIV deaths averted over 10 years. CICF frequencies correspond to screening individuals on average once every five years (CICF frequency per year = 0.2), once every four years (CICF frequency per year = 0.25), once every three years (CICF frequency per year = 0.33), once every two years (CICF frequency per year = 0.5), annually (CICF frequency per year = 1), two times annually (CICF frequency per year = 2), three times annually (CICF frequency per year = 3), four times annually (CICF frequency per year = 4), and five times annually (CICF frequency per year = 5).

To determine the percentage of cases averted by community-based, integrated TB/HIV screening, we modeled TB and HIV incidences and deaths that would occur over 10 years when CICF was implemented at different frequencies, ranging from screening the population on average once every five years to five times annually. We then calculated the percentage reduction of cases or deaths (*i*.*e*., percentage of cases or deaths averted) relative to the status quo (Fig 2). We found that for a CICF frequency of screening individuals on average once every five years, 1% of TB and HIV cases were averted after the first year of CICF implementation, while 6% of TB and 3% of HIV cases were averted after 10 years of implementation (Fig 2A and 2B). For South Africa’s goal of an annual screening frequency, 4% of TB and 5% of HIV cases were averted after one year of CICF implementation (Fig 2A and 2B), and 23% of TB and 12% of HIV cases were averted after 10 years (Fig 2A and 2B), including 24% of drug susceptible, 10% of MDR, and 9% of XDR-TB cases (Fig 2C–2E). For a screening frequency of five times annually, 13% of TB and 15% of HIV cases were averted after the first year of CICF implementation, while 10% of TB and 26% of HIV cases were averted after 10 years (Fig 2A and 2B). We found that for CICF screening frequencies between three times and five times annually, MDR-TB and XDR-TB incidence increased over time and the corresponding percentages of cases averted were reduced (Fig 2D and 2E). These increases were a result of deficiencies in the status quo second-line TB treatment program, as well as reduction in the immunity that would have otherwise been conferred from latent infection with drug susceptible TB. Although drug susceptible TB incidence was reduced as CICF frequency increased, the increase in MDR and XDR-TB incidence with increased CICF frequency resulted in a reduction in the overall percentage of total TB cases averted.

We found that increasing ART eligibility from CD4+ cell count ≤ 350 cells per milliliter to ≤ 500 cells per milliliter reduced the status quo incidences of TB and HIV, as well as increased the percentage of drug susceptible TB cases averted, HIV cases averted, and TB and HIV deaths averted by CICF for all screening frequencies (S2 Fig). MDR and XDR-TB incidence remained high, due to inadequacies in the status quo second-line TB treatment program.

### Comparative Effectiveness of Interventions

To evaluate the effectiveness of CICF relative to the recommended TB/HIV interventions, we projected TB and HIV cases and deaths averted relative to status quo and compared to GeneXpert, MDR-TB treatment decentralization, improved cure rate for first-line TB treatment, IPT, and ART expansion. For this comparison, we considered an annual CICF screening frequency as the goal scale-up of current CICF screening in KwaZulu-Natal [12].

**Fig 3:**
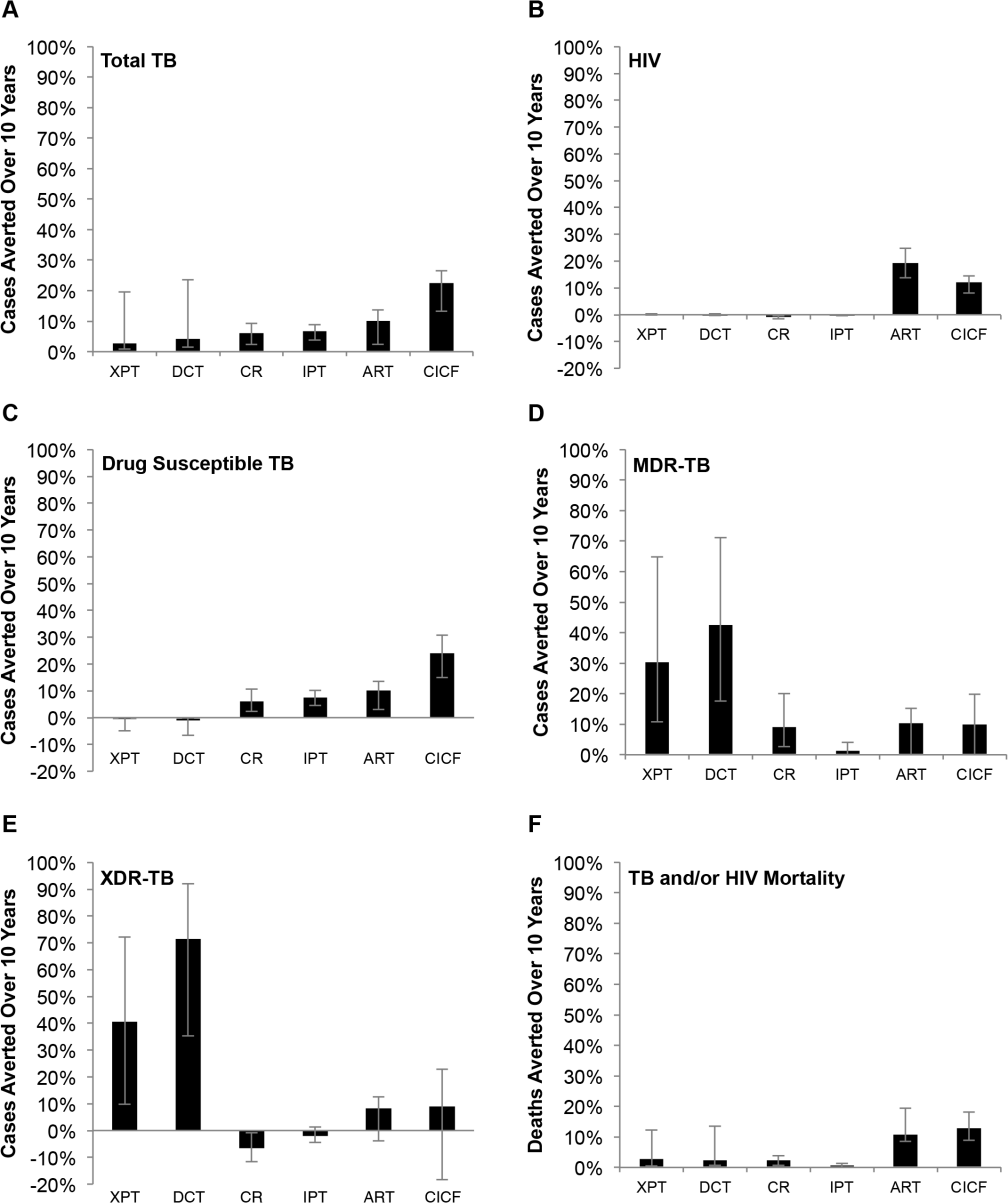
Intervention Impacts. Impact of each intervention implemented by itself on (A) total TB cases averted, (B) HIV infections averted, (C) drug susceptible TB cases averted, (D) MDR-TB cases averted, (E) XDR-TB cases averted, and (F) TB and HIV deaths averted over 10 years. XPT: replacement of sputum smear with GeneXpert for baseline microbiologic TB detection. DCT: MDR-TB treatment decentralization, corresponding to new South African guidelines for MDR-TB care. CR: increased cure rate of first-line treatment to 85% for drug susceptible TB, corresponding to WHO goals. IPT: 36 months of IPT for all TST+ HIV+ individuals on ART and 12 mo. for all TST- HIV+ individuals on ART, corresponding to anticipated implementation of new South African guidelines. ART: increasing rate of ART initiation by 24% annually, culminating in the South African goal of 80% ART coverage by 2016. CICF: annual frequency for TB and HIV screening program CICF.

Each intervention reduced TB and/or HIV incidence, but the impact varied greatly among interventions. Some interventions were more effective at reducing one type of disease than another type of disease, such that no single intervention was simultaneously most effective for drug susceptible TB, MDR-TB, XDR-TB, and HIV. We found that annual CICF, which combines both TB and HIV screening, had the greatest impact on averting TB cases relative to the other interventions, reducing total and drug susceptible TB by 23% (95% CI 13–27%) and 24% (95% CI 15–31%), respectively, (Fig 3A and 3C), and TB/HIV related mortality by 13% (95% CI 9–18%; Fig 3F). Annual CICF also reduced MDR (10% averted; 95% CI—6–20%; Fig 3D) and XDR-TB (9% averted; 95% CI—18–23%; Fig 3E), as well as HIV (12% averted; 95% CI 8–15%; Fig 3B).

Expanding ART coverage was the second most effective TB intervention, averting 10% (95% CI 2–14%) of total TB cases (Fig 3A). Additionally, it averted more HIV infections than any other intervention (19%; 95% CI 14–25%; Fig 3B). Decreasing ART efficacy (corresponding to increasing ART resistance) reduced the percentages of TB and HIV cases that would have been averted by ART (S3 Fig). Reducing ART efficacy to 50% almost halved the percentage of TB cases averted, from 10% to 6%, and quartered the percentage of HIV cases averted, from 19% to 4%. When ART was additionally made available to HIV infected individuals with a CD4+ cell count between 350 and 500 cells per milliliter (assuming an ART coverage equal to that of individuals with a CD4+ cell count ≤ 350) the percentages of TB and HIV cases and deaths averted (including drug susceptible, MDR, and XDR-TB) were doubled (S4 Fig).

IPT of HIV positive individuals on ART averted 7% (95% CI 4–9%) of total and 8% (95% CI 5–10%) of drug susceptible TB (Fig 3A and 3C), but minimally impacted drug resistant TB, HIV, and mortality from TB and/or HIV. Increasing the cure rate of first-line therapy for drug susceptible TB to 85% reduced drug susceptible TB by 6% (95% CI 2–11%; Fig 3C), MDR-TB by 9% (95% CI 3–20%; Fig 3D), and mortality by 3% (95% CI 1–4%; Fig 3F), and negligibly impacted XDR-TB and HIV incidence (Fig 3B and 3E).

The decentralization of MDR-TB treatment had the greatest impact on drug resistant TB overall, reducing MDR-TB by 43% (95% CI 18–71%; Fig 3D) and XDR-TB by 72% (95% CI 35–92%; Fig 3D). However, it had little impact on total TB or HIV (Fig 3B, 3C and 3F). Due to increased detection and subsequent treatment of MDR and XDR-TB, GeneXpert achieved the second most significant reduction in MDR-TB (31% averted; 95% CI 11–65%; Fig 3D and XDR-TB (41% averted; 95% CI 10–72%; Fig 3E), in addition to negligible effects on drug susceptible TB and HIV ((Fig 3B, 3C and 3F).

Overall, annual CICF was most effective at reducing total TB cases and disease-related mortality, ART had the greatest impact on HIV infections, and decentralized MDR-TB treatment and GeneXpert combined with DST most significantly reduced MDR and XDR-TB cases.

### Combining Interventions

**Fig 4:**
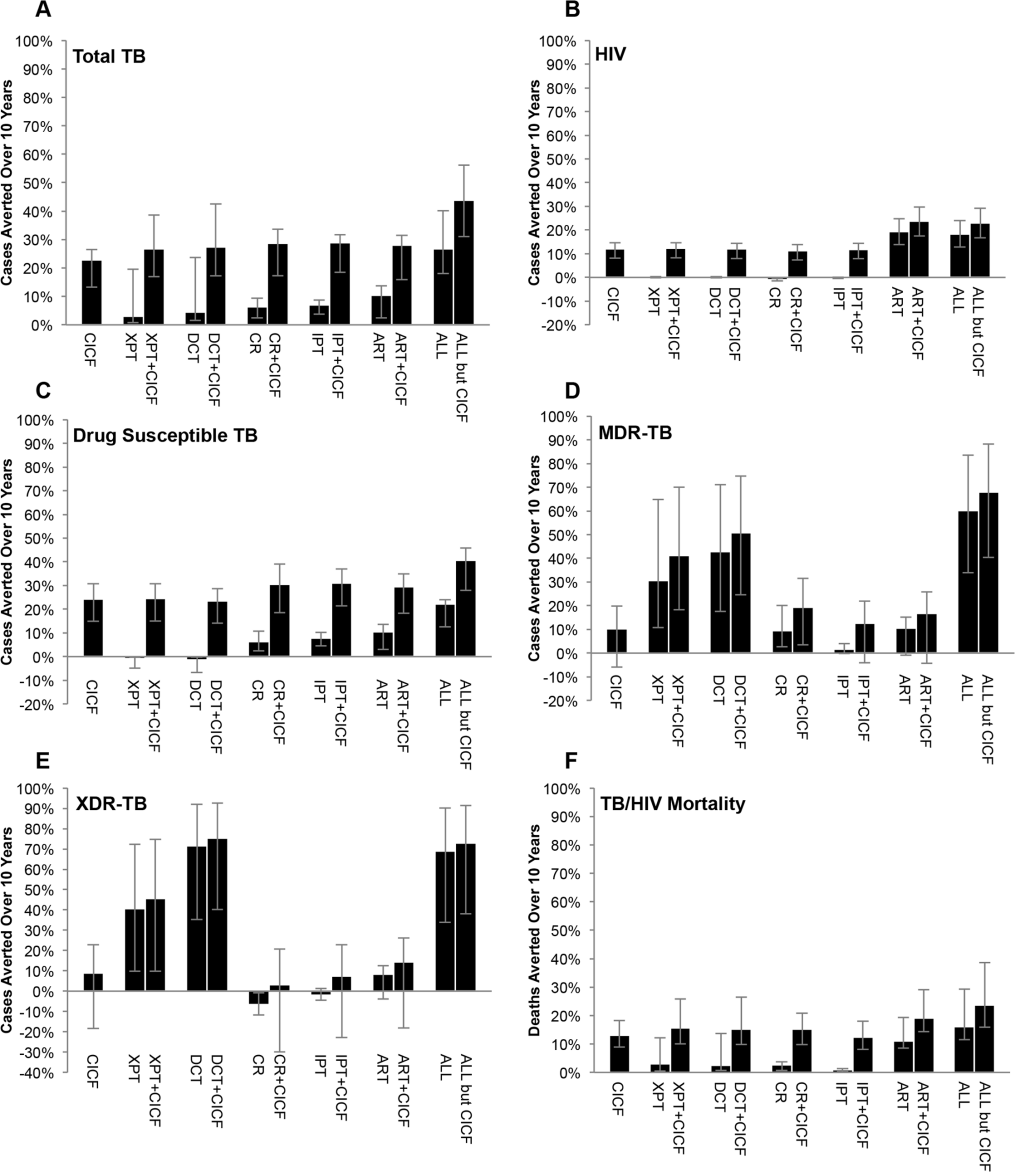
Combined Intervention Impacts. Impact of interventions integrated with an annual CICF frequency on (A) total TB cases averted, (B) HIV infections averted, (C) drug susceptible TB cases averted, (D) MDR-TB cases averted, (E) XDR-TB cases averted, and (F) TB and HIV deaths averted over 10 years. XPT: replacement of sputum smear with GeneXpert for baseline microbiologic TB detection. DCT: MDR-TB treatment decentralization, corresponding to new South African guidelines for MDR-TB care. CR: increase cure rate of first-line treatment to 85% for drug susceptible TB, corresponding to WHO goals. IPT: 36 months of IPT for all TST+ HIV+ individuals on ART and 12 mo. for all TST—HIV+ individuals on ART, corresponding to anticipated implementation of new South African guidelines. ART: increasing rate of ART initiation by 24% annually, culminating in the South African goal of 80% ART coverage by 2016. CICF: annual frequency for TB and HIV screening program CICF. ALL but CICF: XPT, DCT, CR, IPT, and ART combined. ALL: XPT, DCT, CR, IPT, ART, and CICF combined.

Our analysis of the comparative effectiveness of CICF and recommended TB/HIV interventions showed that the addition of annual CICF to the other interventions averted additional morbidity and mortality over 10 years, compared to the status quo of screening and treatment implemented (Fig 4A–4F). We considered both the contribution of annual CICF to each recommended intervention individually and to all the recommended interventions combined. Adding annual CICF to recommended interventions individually averted an additional 19–26% of drug susceptible TB cases (Fig 4C), 6–11% of MDR-TB cases (Fig 4D), 3–10% of XDR-TB cases (Fig 4E), 5–12% of HIV infections (Fig 4B), and 8–13% of TB/HIV deaths (Fig 4F) over 10 years.

Combining recommended interventions in the absence of annual CICF averted approximately 27% (95% CI 18–40%) of expected TB cases, 18% (95% CI 13–24%) of HIV infections, 60% (95% CI 34–83%) of MDR-TB cases, 69% (95% CI 34–90%) of XDR-TB cases, and 16% (95% CI 12–29%) of TB and/or HIV deaths over 10 years (Fig 4). The addition of annual CICF to this combination averted a further 19% of drug susceptible TB cases (Fig 4C), 8% of MDR-TB cases (Fig 4D), 4% of XDR-TB cases (Fig 4E), 5% of HIV infections (Fig 4B), and 8% of TB/HIV deaths (Fig 4F). When all interventions were combined, 44% (95% CI 31–56%) of total TB cases were averted and 23% (95% CI 17–29%) of HIV cases, with 41% (95% CI 28–46%) of drug susceptible TB cases, 68% (95% CI 40–88%) of MDR-TB cases, 73% (95% CI 38–91%) of XDR-TB cases, and 24% (95% CI 16–39%) of TB and/or HIV deaths averted.

**Fig 5:**
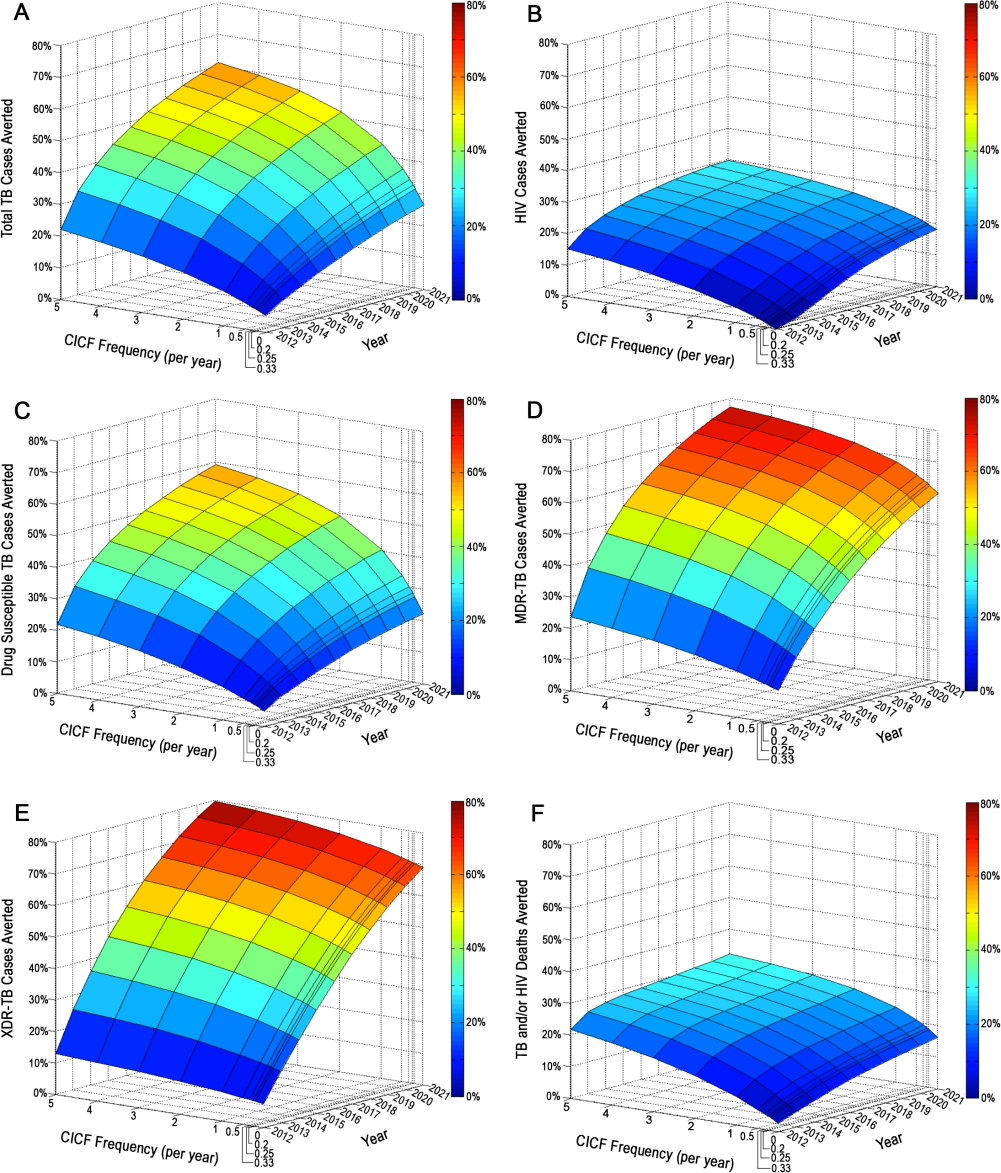
Combined Intervention Impacts with Variable CICF Screening Frequency. The impact of CICF at different screening frequencies combined with GeneXpert, MDR-TB treatment decentralization, improved drug susceptible TB cure rate, IPT, and expanded ART over a 10 year period on (A) total TB cases averted, (B) HIV infections averted, (C) drug susceptible TB cases averted, (D) MDR-TB cases averted, (E) XDR-TB cases averted, and (F) TB and HIV deaths averted over 10 years. CICF frequencies correspond to screening individuals on average once every five years (CICF frequency per year = 0.2), once every four years (CICF frequency per year = 0.25), once every three years (CICF frequency per year = 0.33), once every two years (CICF frequency per year = 0.5), annually (CICF frequency per year = 1), two times annually (CICF frequency per year = 2), three times annually (CICF frequency per year = 3), four times annually (CICF frequency per year = 4), and five times annually (CICF frequency per year = 5).

More frequent CICF screening of five times annually additionally improved the impact of the combined interventions on the TB and HIV epidemics, averting 61% of total TB cases (Fig 5A), 30% of HIV cases (Fig 5B), 59% of drug susceptible TB cases (Fig 5C), 77% of MDR-TB cases (Fig 5D), 80% of XDR-TB cases (Fig 5E), and 32% of TB and/or HIV deaths (Fig 5F).

### Sensitivity Analysis

We conducted a global sensitivity analysis of model parameters and outcomes to determine which parameters have the most significant impact on our evaluations of CICF (S3 Table). Sensitivity of the symptom questionnaire was identified as the most influential CICF-specific parameter; improving the sensitivity of the symptom questionnaire increased the number of TB cases that could be detected and averted by CICF. Further, greater effectiveness of CICF was most strongly associated with shortening the delay until diagnosis and treatment, efficacies of second-line TB treatment, and reducing default before TB treatment initiation, particularly in averting MDR and XDR-TB. Default before initiating ART was identified as the most influential HIV control parameter.

An increase in the status quo TB detection and treatment rate while implementing CICF was correlated with a reduction in the percentage of drug susceptible TB and HIV cases averted, but an increase in the percentage of MDR and XDR-TB cases averted by CICF. Parameters associated with increasing the status quo TB incidence—including increasing the force of infection for TB or the proportion of individuals who quickly reactivate from latent to infectious active TB disease, but not the rate at which individuals progress from noninfectious to infectious active TB disease—were also correlated with an increase in the percentage of TB cases averted by CICF. An increase in the status quo HIV detection and treatment rate was associated with a reduction in the percentage of all TB and HIV cases averted by CICF.

## Discussion

We evaluated the impact of CICF, a community-based, integrated TB/HIV screening program that additionally links individuals to treatment of both TB and HIV, on the convergent TB/HIV epidemics in rural South Africa. We compared the impact of CICF to that of an array of TB and HIV interventions, including GeneXpert, decentralization of MDR-TB treatment, improved first-line TB treatment cure rate, IPT, and expanded ART coverage. We found that alone and in combination with recommended TB and HIV interventions, CICF contributed substantially to TB/HIV control. CICF successfully reduced TB and HIV incidence and mortality, while overlapping very little with most recommended interventions. Our findings indicate that CICF combined with recommended TB/HIV interventions represents a control strategy which comprehensively targets drug susceptible and drug resistant TB and HIV, and can successfully address the TB/HIV co-epidemics in South Africa. Although South Africa aims to conduct annual screening, the actual achievable frequency of screening in a given population may depend on local resource constraints and other practical limitations.

Our analysis showed that CICF successfully reduces TB and HIV incidence and mortality via the detection of cases, the identification of individuals who are eligible for treatment (including preventive treatment), and the linkage of these individuals to the appropriate treatment. When implemented alone, relative to the other recommended interventions (*i*.*e*., GeneXpert, MDR-TB care decentralization, improved drug susceptible TB cure rate, IPT, and ART expansion), we saw that annual CICF had the greatest reduction in both drug susceptible TB incidence and TB and/or HIV mortality, as well as the second greatest reduction in HIV cases, relative to the other modeled interventions. Annual CICF also reduced the number of MDR and XDR-TB cases, but not as effectively as GeneXpert or decentralizing MDR-TB treatment. Even greater reductions in drug susceptible TB incidence and TB and/or HIV mortality were observed when CICF frequency was increased up to five times per year (on average), thereby increasing the number of TB and HIV cases detected. Increases in both the number of drug susceptible and drug resistant TB cases detected by CICF could also be achieved if the sensitivity of CICF’s TB symptom questionnaire was improved or the sensitivity of the tool used by CICF for detecting MTB in sputum was improved (*e*.*g*., sputum smear replaced with GeneXpert). Reductions in MDR and XDR-TB were not observed at very high frequencies of CICF (*i*.*e*., screening three times or more per year) due to second-line TB treatment initiation delays, default, and low cure rates. Improving second-line treatment delays, default, or cure rates—including combining CICF with GeneXpert and/or decentralized MDR-TB treatment—could reverse the effect of high CICF frequency on reducing the number of MDR and XDR-TB cases and further improve CICF’s reduction of drug resistant TB incidence at lower screening frequencies. Increasing first-line TB treatment cure rates also increased the number of drug susceptible TB cases averted by CICF.

When all interventions were implemented in combination (*i*.*e*., CICF plus GeneXpert, MDR-TB care decentralization, improved drug susceptible TB cure rate, IPT, and ART expansion), TB and HIV incidence and mortality were further reduced. These reductions were not only the result of increases in the number of drug susceptible, MDR, and XDR-TB and HIV cases detected and linked to treatment by CICF plus GeneXpert, but also a result of the increase in the first-line TB treatment cure rate, decrease in default from second-line TB treatment (due to MDR-TB treatment decentralization), decrease in delay between drug resistant TB diagnosis and second-line TB treatment initiation (due to MDR-TB treatment decentralization), and increase in the number of HIV infected individuals on both ART and IPT. ART expansion also reduced TB and HIV incidence and mortality. However, its impact on HIV incidence, when combined with CICF, was mitigated by the fact that CICF also increases ART coverage. Thus, both interventions reduce HIV through the same mechanism.

Our model is the first to examine the impact of GeneXpert, MDR-TB treatment decentralization, improved first-line TB treatment cure rate, IPT, ART expansion, and CICF in combination on the TB/HIV epidemics in rural South Africa, as well as the first to evaluate the effects of CICF alone. We confirmed that increased TB case detection (*i*.*e*., a community TB/HIV screening program), prevention (*i*.*e*., IPT), and increasing cure rates are most effective at reducing total TB in rural South Africa [3,11,15,16,22,41,66,67]. We additionally confirmed that the impact of IPT was greatest for drug susceptible TB, rather than MDR or XDR-TB [24,68], but found a lower effect relative to previous predictions. The difference compared to other predictions arises from our consideration of the observed practice that only individuals on ART currently receive IPT [5]. Expanding IPT access to HIV infected individuals who are not yet eligible for ART is likely to further reduce drug susceptible TB incidence. Unlike other studies, we also demonstrated the effects of the above TB-focused interventions on the HIV epidemic. Specifically, we found that TB interventions alone extend the lifespan of HIV infected individuals (reducing mortality), but slightly increase HIV incidence because there is no reduction in HIV viral load or infectiousness. We confirmed that ART successfully reduces both TB and HIV incidence and mortality, although the reductions we observed were not as great as those observed in other studies [16,23]. This discrepancy is explained by the fact that other studies assume a near 100% coverage of ART for HIV infected individuals, while we assumed more realistic levels of ART coverage (*i*.*e*., 52% status quo and 80% when expanded) and default (*i*.*e*., 75% three year retention status quo and 70% five year retention when expanded). We demonstrated the reductions in MDR and XDR-TB incidence that could be achieved with the decentralization of MDR-TB treatment, something that has yet to be explored by studies on a population level. We also confirmed that GeneXpert successfully reduces the incidence of MDR-TB and XDR-TB. Like recent studies [58,59], we found that GeneXpert had limited impact on drug susceptible TB incidence and mortality, due to the common practice of empirical clinical diagnosis of TB in resource-limited settings. We recognize that GeneXpert may have a greater impact over time as the technology becomes better incorporated into clinical practice [59].

Our study supports the full integration of TB and HIV control, which has been recommended by the WHO [5,6], but is still not comprehensively implemented in many countries [5,6] and has not yet been explicitly evaluated [26]. We found that integrated interventions are necessary to effectively address the colliding epidemics of drug susceptible TB, MDR-TB, XDR-TB, and HIV. In fact, in the absence of integration, an intervention may improve the incidence of one disease while negligibly impacting or even slightly exacerbating another. For example, we saw that TB-only interventions reduced TB incidence but slightly increased HIV incidence as a result of increases in the lifespans of HIV positive individuals without reductions in HIV transmission. Integration is not only important for TB and HIV control, but also for drug susceptible and drug resistant TB control. This is evidenced by the negligible impact of drug resistant TB-focused interventions on drug susceptible TB incidence, and vice versa. Additionally, we found that even when an intervention, such as CICF, focuses on drug resistant TB, limitations in the second-line TB treatment programs (*e*.*g*., lower drug efficacy, delays to diagnosis and treatment, and high default before initiating treatment) can exacerbate MDR or XDR-TB incidence. This increased incidence is due to increased amplification of drug resistance arising from TB treatment failures and ongoing transmission of drug resistant TB, as well as to reduced immunity conferred by latent infection with drug susceptible TB.

Mathematical modeling allowed us to estimate the combined effects of these TB and HIV interventions on the joint TB/HIV epidemics. As with any modeling study, simplifying assumptions were required to model the transmission of two diseases. Although we explicitly modeled the dynamics of the TB/HIV co-epidemics and accounted for HIV prevalence-dependent behavior change, we did not include additional HIV risk reduction strategies. Although detection and ART are the primary approaches to control HIV [8], additional control strategies would be expected to decrease TB and HIV incidence and mortality further [69]. All modeling studies are also limited by the empirical uncertainty of the data used to construct and parameterize the model. Furthermore, South Africa’s approach to TB and HIV control is rapidly evolving, and the current status quo will not be the same in the future. We accounted for empirical uncertainty and its impact on model outcomes via uncertainty analysis and the construction of confidence intervals on model outcomes. We additionally used sensitivity analysis to further examine the robustness of the model predictions. We found that increases in the status quo TB detection and treatment rate reduced the percentage of drug susceptible TB cases averted by annual CICF, because there were fewer TB cases overall. The percentages of MDR-TB, XDR-TB, and HIV cases averted by CICF were higher when the status quo TB detection and treatment rates were increased, because there were a larger number of cases at status quo to avert. MDR and XDR-TB incidence increased because, without additional measures to control drug resistant TB, higher rates of first-line TB treatment generated additional cases of MDR and XDR-TB. Similarly, HIV incidence increased because, in the absence of additional HIV control measures, individuals infected with HIV will live longer when TB transmission is reduced and therefore be able to transmit HIV for a longer period of time. We also found that increases in the status quo HIV detection and treatment rate reduced the percentage of TB and HIV cases averted by annual CICF, because there were fewer TB and HIV cases overall.

In conclusion, we found that the convergent TB/HIV epidemics in South Africa were most effectively curtailed by simultaneously implementing interventions that integrated community-based TB/HIV control strategies, as well as by simultaneously targeting drug-resistant TB. CICF, a screening program that conducts TB/HIV case finding in the community, while also linking patients to treatment, conferred additional reductions in disease incidence and mortality when combined with recommended TB and HIV interventions. The data from this study support the idea that in order to reduce the morbidity and mortality of the convergent epidemics of drug susceptible and drug resistant TB and HIV in the coming decade, TB and HIV interventions must be more completely integrated, and strengthened with the addition of a community-based integrated TB/HIV screening program that actively links identified cases to treatment.

## Supporting Information

S1 Fig Model Calibration and Validation. Model calibration and validation using (A) TB incidence, (B) TB prevalence, and (C) HIV prevalence data. Error bars indicate available minimum and maximum values in data.
(PDF)

S2 Fig CICF with ART eligibility at CD4+ cell count ≤ 500 cells per milliliter. Impact of increasing eligibility to initiate ART from CD4+ cell count ≤ 350 cells per milliliter to a CD4+ cell count ≤ 500 cells per milliliter, and assuming ART coverage for individuals with a CD4+ cell count between 350 and 500 cells per milliliter is equal to the ART coverage for individuals with a CD4+ cell count below 350 cells per milliliter, on total TB cases averted, HIV infections averted, drug susceptible TB (DS TB) cases averted, MDR-TB cases averted, XDR-TB cases averted, and TB/HIV deaths averted by CICF at frequencies corresponding to screening individuals on average once every five years (CICF frequency per year = 0.20), once every four years (CICF frequency per year = 0.25), once every three years (CICF frequency per year = 0.33), once every two years (CICF frequency per year = 0.5), annually (CICF frequency per year = 1), two times annually (CICF frequency per year = 2), three times annually (CICF frequency per year = 3), four times annually (CICF frequency per year = 4), and five times annually (CICF frequency per year = 5).
(PDF)

S3 Fig Decreased ART Efficacy. Impact of decreasing ART efficacy on total TB cases averted, HIV infections averted, drug susceptible TB (DS TB) cases averted, MDR-TB cases averted, XDR-TB cases averted, and TB/HIV deaths averted by expanding ART coverage to 80% and increasing five-year retention to 70% by 2016. Note that Total TB and DS TB lines overlap because they are very close in value; the majority of total TB cases are drug susceptible.
(PDF)

S4 Fig Expansion of ART coverage with ART eligibility at CD4+ cell count ≤ 500 cells per milliliter. Impact of increasing eligibility to initiate ART from CD4+ cell count ≤ 350 cells per milliliter to a CD4+ cell count ≤ 500 cells per milliliter, with ART coverage for individuals with a CD4+ cell count between 350 and 500 cells per milliliter varied between 0% and 100% of the ART coverage for individuals with a CD4+ cell count ≤ 350 cells per milliliter, on total TB cases averted, HIV infections averted, drug susceptible TB (DS TB) cases averted, MDR-TB cases averted, XDR-TB cases averted, and TB/HIV deaths averted by expanding ART coverage to 80% and increasing five-year retention to 70% by 2016. Note that Total TB and DS TB lines overlap because they are very close in value; the majority of total TB cases are drug susceptible.
(PDF)

S1 Table Model parameters, ranges, and references. Rates are in units of year^-1^ unless otherwise noted. *HIV+ individuals on ART have the effect of HIV on the natural history of TB reduced by the effectiveness of ART (parameter *treat*).
(PDF)

S2 Table Additional Model Calibration and Validation. (PDF)

S3 Table Global Sensitivity Analysis. Partial rank correlation coefficients (PRCCs) and p-values calculated from model outcomes generated from minimum and maximum of parameter distributions found in S1 Table in a global sensitivity analysis of annual CICF screening.
(PDF)

S1 Text Technical Appendix. Supplementary figures and tables are available as individual files, but are also included here for convenience, along with additional model details.
(PDF)
