# Supplementary figures and images for: Integrating Community-Based Interventions to Reverse the Convergent TB/HIV Epidemics in Rural South Africa
Source: PLoS One. 2015 May 4;10(5):e0126267. doi: 10.1371/journal.pone.0126267 (PMC4418809; doi:10.1371/journal.pone.0126267)

**A****TB Incidence**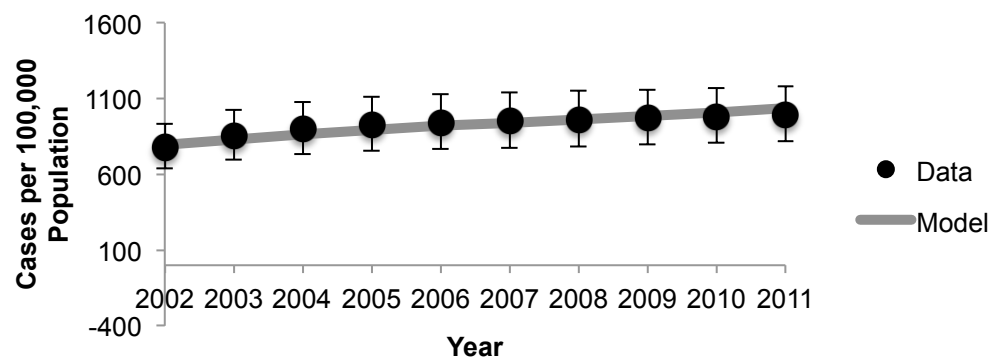**B****TB Prevalence**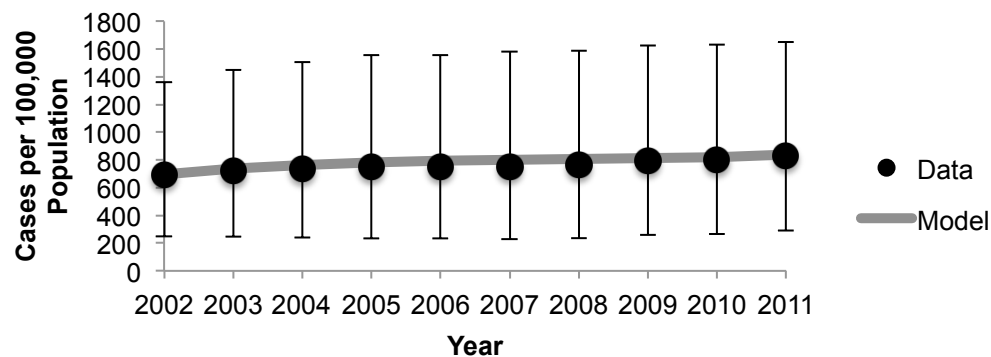**C****HIV Prevalence**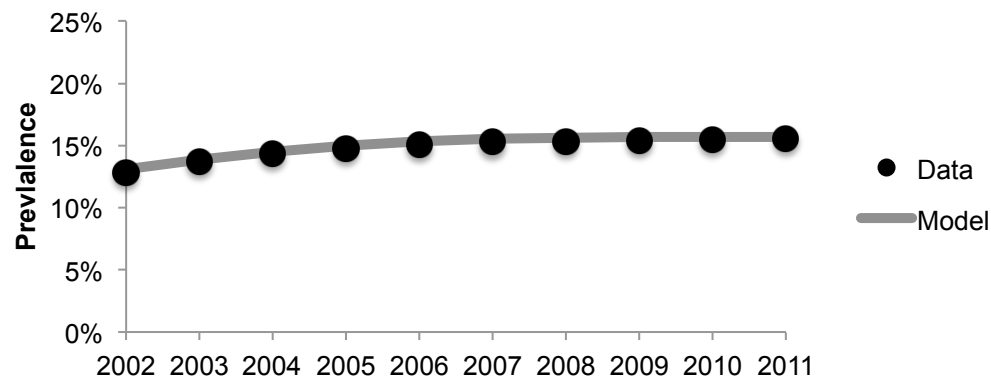

Supplement: S1 Fig — Model calibration and validation using (A) TB incidence, (B) TB prevalence, and (C) HIV prevalence data. Error bars indicate available minimum and maximum values in data. (PDF) [file pone.0126267.s001.pdf]

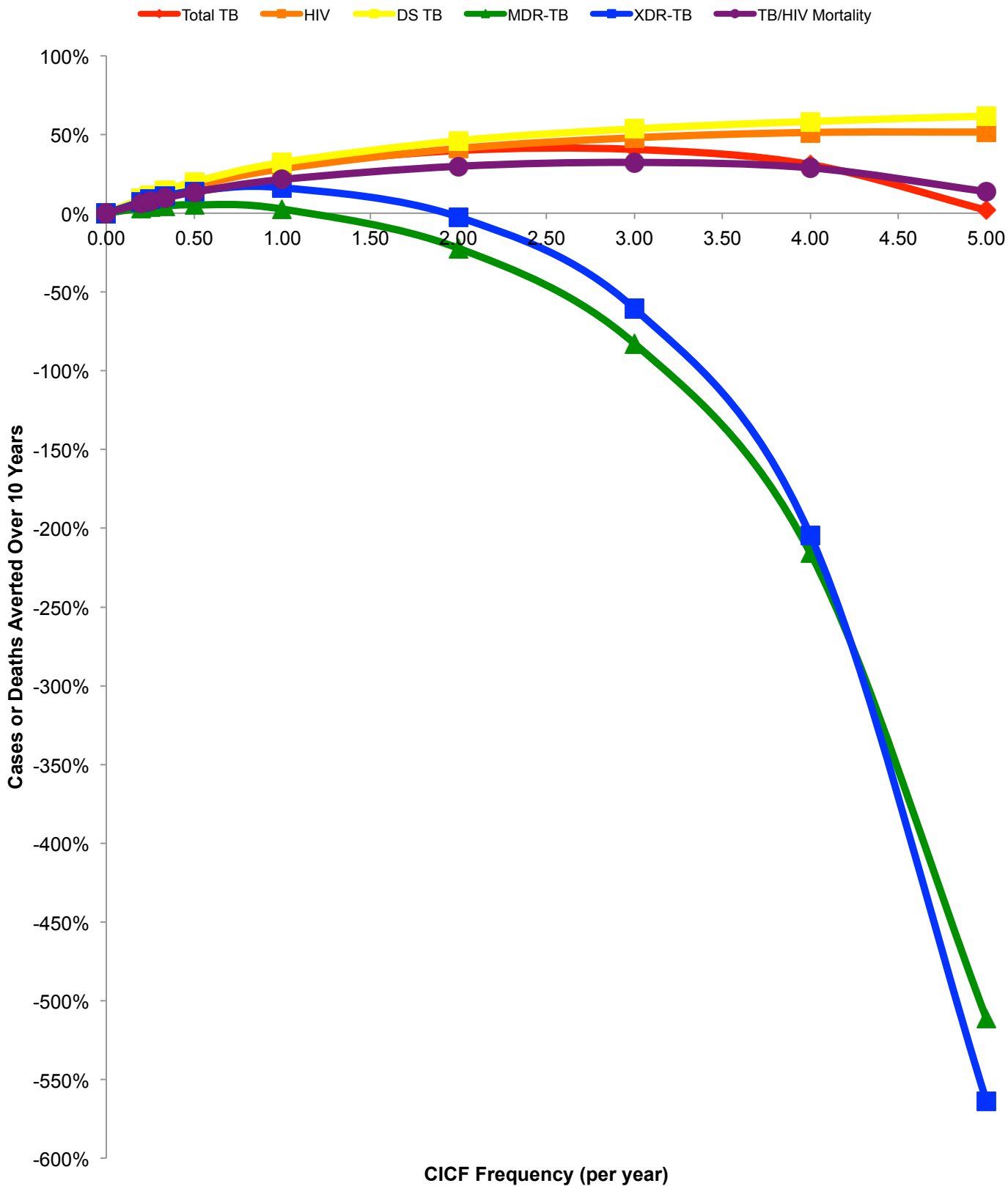

Supplement: S2 Fig — Impact of increasing eligibility to initiate ART from CD4+ cell count ≤ 350 cells per milliliter to a CD4+ cell count ≤ 500 cells per milliliter, and assuming ART coverage for individuals with a CD4+ cell count between 350 and 500 cells per milliliter is equal to the ART coverage for individuals with a CD4+ cell count below 350 cells per milliliter, on total TB cases averted, HIV infections averted, drug susceptible TB (DS TB) cases averted, MDR-TB cases averted, XDR-TB cases averted, and TB/HIV deaths averted by CICF at frequencies corresponding to screening individuals on average once every five years (CICF frequency per year = 0.20), once every four years (CICF frequency per year = 0.25), once every three years (CICF frequency per year = 0.33), once every two years (CICF frequency per year = 0.5), annually (CICF frequency per year = 1), two times annually (CICF frequency per year = 2), three times annually (CICF frequency per year = 3), four times annually (CICF frequency per year = 4), and five times annually (CICF frequency per year = 5). (PDF) [file pone.0126267.s002.pdf]

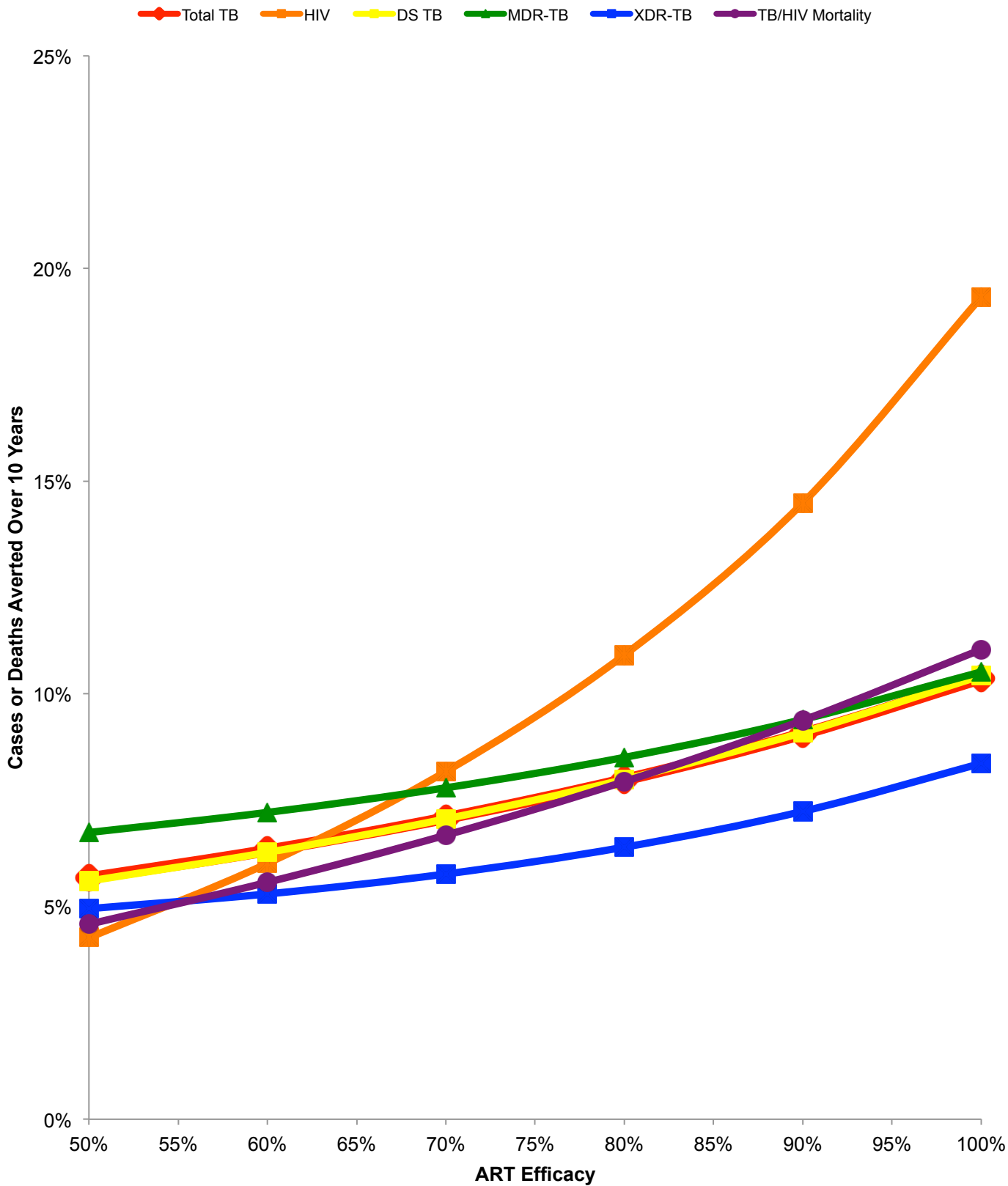

Supplement: S3 Fig — Impact of decreasing ART efficacy on total TB cases averted, HIV infections averted, drug susceptible TB (DS TB) cases averted, MDR-TB cases averted, XDR-TB cases averted, and TB/HIV deaths averted by expanding ART coverage to 80% and increasing five-year retention to 70% by 2016. Note that Total TB and DS TB lines overlap because they are very close in value; the majority of total TB cases are drug susceptible. (PDF) [file pone.0126267.s003.pdf]

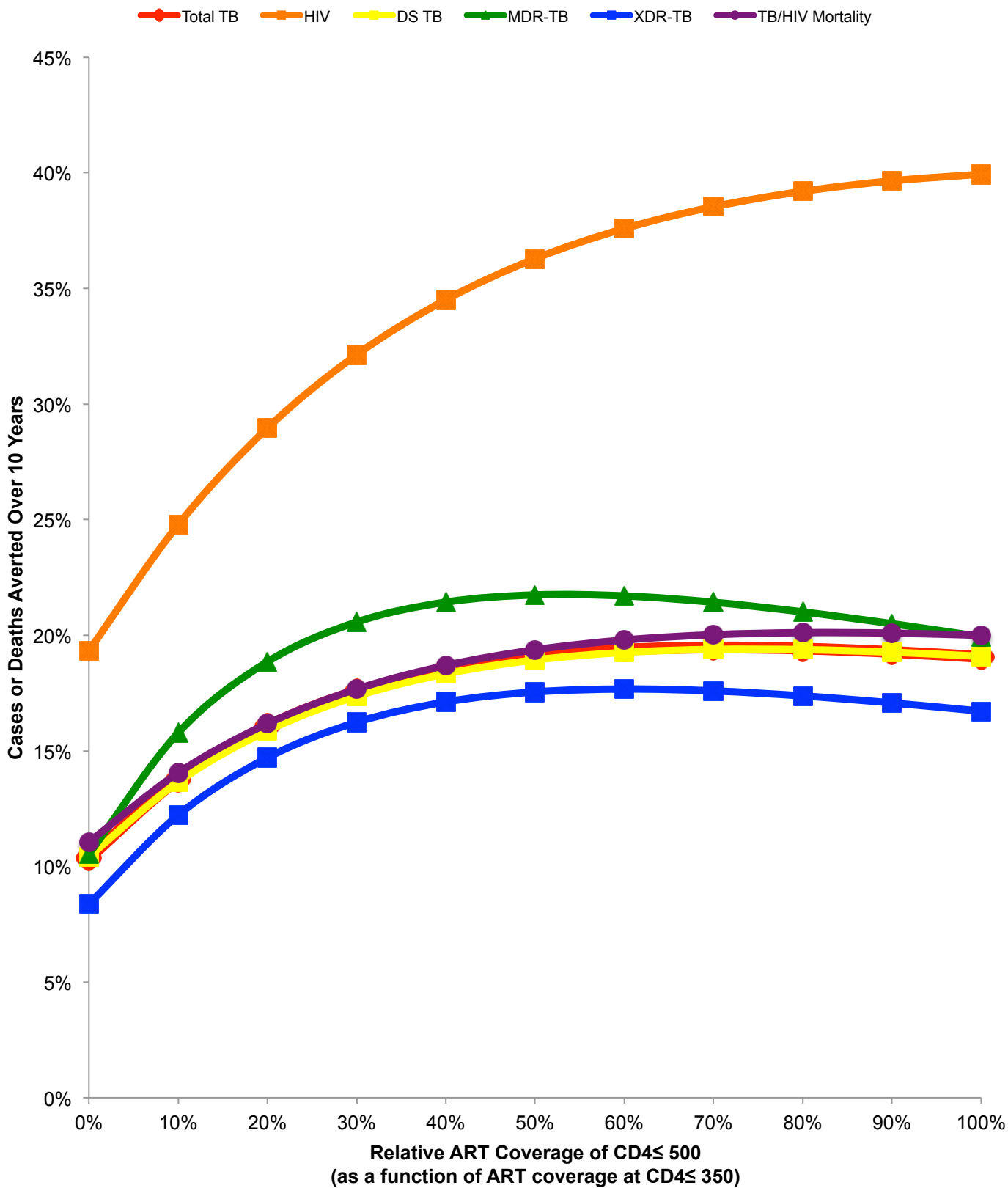

Supplement: S4 Fig — Impact of increasing eligibility to initiate ART from CD4+ cell count ≤ 350 cells per milliliter to a CD4+ cell count ≤ 500 cells per milliliter, with ART coverage for individuals with a CD4+ cell count between 350 and 500 cells per milliliter varied between 0% and 100% of the ART coverage for individuals with a CD4+ cell count ≤ 350 cells per milliliter, on total TB cases averted, HIV infections averted, drug susceptible TB (DS TB) cases averted, MDR-TB cases averted, XDR-TB cases averted, and TB/HIV deaths averted by expanding ART coverage to 80% and increasing five-year retention to 70% by 2016. Note that Total TB and DS TB lines overlap because they are very close in value; the majority of total TB cases are drug susceptible. (PDF) [file pone.0126267.s004.pdf]
