# Supplementary material for: Integrating Community-Based Interventions to Reverse the Convergent TB/HIV Epidemics in Rural South Africa
Source: PLoS One. 2015 May 4;10(5):e0126267. doi: 10.1371/journal.pone.0126267 (PMC4418809; doi:10.1371/journal.pone.0126267)
Supplement: S1 Table — Rates are in units of year-1 unless otherwise noted. *HIV+ individuals on ART have the effect of HIV on the natural history of TB reduced by the effectiveness of ART (parameter treat). (PDF) [file pone.0126267.s005.pdf]

S1 Table. Model parameters, ranges, and references. Rates are in units of year^{-1} unless otherwise noted. *HIV+ individuals on ART have the effect of HIV on the natural history of TB reduced by the effectiveness of ART (parameter *treat*).

Parameter	Symbol	TB Disease Status	HIV–	HIV+ (No ART)	HIV+ (ART)	Reference
**Annual population growth rate**	g		0.0572 (0.02–0.06)	NA	NA	Fitted to [1,2]
**Annual mortality rate**	μ	*Baseline (no TB)*	0.003 (0.0009–0.004)	0.0155 (0.004–0.13, CD4+ >350), 0.339 (0.21–0.64, CD4+ ≤350)	0.0155 (0.004–0.13, CD4+ >350), 0.122 (0.004–0.13, CD4+ ≤350)	Fitted to [1,2], ranges adapted from [3-8]
	μ_{TB}	*Active TB*	0.30 (0.2–0.4)	0.992 (0.75–1)	0.508 (0.2–0.76)	Fitted to [1,2], ranges adapted from [3]
**TB transmissibility coefficient**	β_{TB}		9.52 x 10^{-5}	9.52 x 10^{-5}	9.52 x 10^{-5}	Fitted to [1,2]
**Relative fitness penalty drug resistance TB (proportion)**	fit_2	*MDR*	0.12 (0–0.7)	0.12 (0–0.7)	0.12 (0–0.7)	[3,9]
	fit_3	*XDR*	0.16 (0–0.7)	0.16 (–-0.7)	0.16 (0–0.7)	[3,9]
**Proportion who develop primary progressive TB**	p		0.14 (0.08–0.25)	0.67 (0.36–0.8)	*	[3,10]
**Endogenous annual TB reactivation rate**	ν	*Long latency*	0.00011 (0.0001–0.0003)	0.17 (0.04–0.2)	*	[3,10]
	τ	*Short latency*	0.88 (0.76–0.99)	12 (10.4–13.5)	*	[3,10]
**Degree susceptibility despite prior TB infection (proportion)**	x_{TB}		0.35 (0.1–0.6)	0.75 (0.5–1)	*	[3]
**Proportion of TB infections that are infectious**	f		0.65 (0.5–0.65)	0.3 (0.19–0.4)	*	[3]
**Rate of conversion from non-infectious to infectious TB**	w		0.015 (0.007–0.02)	0.015 (0.007–0.02)	0.015 (0.007–0.02)	[3,11]
**Rate of natural self cure**	ξ		0.2 (0.15–0.25)	0.2 (0.15–0.25)	0.2 (0.15–0.25)	[3]
**Baseline TB detection and treatment**	δ		1.0299 (0.8415–1.1137)	1.0299 (0.8415–1.1137)	1.0299 (0.8415–1.1137)	Fitted to [1,2]
**Proportion cured by 1st line TB drugs**	q_{11}	DS	0.73 (0.65–0.88)	0.58 (0.52–0.74)	*	[3,9]
	q_{12}	MDR	0.47 (0.18–0.58)	0.30 (0.16–0.36)	*	[3,9,12,13]
	q_{13}	XDR	0	0	0	[3,9,12,13]
**Proportion cured by 2nd line TB drugs**	q_{22}	MDR	0.67 (0.43–0.80)	0.45 (0.43–0.51)	*	[3,9,12-14]
	q_{33}	XDR	0.54 (0.328–0.545)	0.36 (0.328–0.400)	*	[3,12,15]
**Duration TB treatment (months)**	$\frac{1}{\rho_1}$	DS	6 (6–8)	6 (6–8)	6 (6–8)	[16]
	$\frac{1}{\rho_2}$	MDR	24 (18–24)	24 (18–24)	24 (18–24)	[17]
	$\frac{1}{\rho_3}$	XDR	26 (24.6–27.8)	26 (24.6–27.8)	26 (24.6–27.8)	[10]
**Proportion defaulting from TB treatment**	d_{TB1}	DS	0.07 (0.048–0.088)	0.07 (0.048–0.088)	0.07 (0.048–0.088)	[18]
	d_{TB2}	MDR	0.2 (0.19–0.37)	0.2 (0.19–0.37)	0.2 (0.19–0.37)	[19-21]
	d_{TB3}	XDR	0.28 (0.19–0.37)	0.28 (0.19–0.37)	0.28 (0.19–0.37)	[17,19,22]

**Proportion of treated TB patients who acquire or amplify resistance**	ω_1	*DS to MDR*	0.038 (0.025–0.1)	0.038 (0.025–0.1)	0.038 (0.025–0.1)	[3]
	ω_2	*MDR to XDR*	0.030 (0.025–0.1)	0.030 (0.025–0.1)	0.030 (0.025–0.1)	[3]
**Time to identification of TB treatment failure (months)**	$\frac{1}{\kappa}$		2 (1–4)	2 (1–4)	2 (1–4)	[10]
**Time from culture collection to MDR treatment initiation (days)**	$\frac{1}{\sigma_{DR}}$	*Baseline*	93 (71–120)	93 (71–120)	93 (71–120)	[23]
	$\frac{1}{\sigma_{DS}}$	*Decentralized*	72 (56–99)	72 (56–99)	72 (56–99)	[23]
**Proportion of cases defaulting before TB treatment initiation**	ϕ_{TB}	*DS outpatient (DOTS)*	0.15 (0–0.24)	0.15 (0–0.24)	0.15 (0–0.24)	[24]
		MDR inpatient (baseline)	0.5 (0.29–0.73)	0.5 (0.29–0.73)	0.5 (0.29–0.73)	[17]
		XDR inpatient	0.5 (0.29–0.73)	0.5 (0.29–0.73)	0.5 (0.29–0.73)	[17]
**HIV transmissibility coefficient**	β_{HIV}		NA	1.63×10^{-6}	1.63×10^{-6}	Fitted to [1,2]
**α (HIV transmission shape parameter)**	α		NA	4.55 (1–6)	4.55 (1–6)	Fitted to [1,2]
**Relative infectivity of HIV on ART (proportion)**	x_{HIV}		NA	NA	0.12 (0–0.12)	[25]
**Relative infectivity of ART eligible**	vl		NA	6.87 (1.00–3.33)	NA	Fitted to [1,2], Range from [26]
**ART coverage (CD4+ < 350)**	η		NA	50% (46–57%)	NA	[27]
**Proportion of cases detected defaulting before HIV treatment initiation**	ϕ_{HIV}		NA	0.38 (0.16–0.61)	NA	[28,29]
**Proportion retained in HIV treatment over 3 years**	d_{HIV}		NA	NA	0.75 (0.64–0.87)	[30]
**Rate of relapse from chemotherapeutic cure**	υ		0.001 (0–0.01)	0.001 (0–0.01)	0.001 (0–0.01)	[3]
**Duration of HIV infection, CD4+ cell count >350 (years)**	$\frac{1}{\gamma}$		NA	6 (5–7); 3 (2–4, active TB infx)	NA	[5-8]
**Sensitivity of TB symptom screening**	sx		69% (51.9–83.7%)	79% (58.3–90.9%)	79% (58.3–90.9%)	[31,32]
**Sensitivity of TB sputum smear**	sm_i	*Infectious*	83.3% (80–83.3%)	83.3% (80–83.3%)	83.3% (80–83.3%)	[3,33,34]
	sm_n	*Noninfectious*	0%	0%	0%	[3,33,34]
**Sensitivity of TB sputum culture/DST**	c_i	*Infectious*	100%	100%	100%	[4]
	c_n	*Noninfectious*	68%	68%	68%	[4]
**Sensitivity of Xpert MTB/RIF for TB diagnosis**	sm_{XPTi}	*Infectious*	98.3% (97–99%)	98.3% (97–99%)	98.3% (97–99%)	[35]
	sm_{XPTn}	*Noninfectious*	76.9% (72.4–80.8%)	76.9% (72.4–80.8%)	76.9% (72.4–80.8%)	[35]
**Sensitivity of Xpert MTB/RIF for RIF resistance detection given MTB detected**	xpt		94.4% (90.8–98.6%)	94.4% (90.8–98.6%)	94.4% (90.8–98.6%)	[35]
**Sensitivity of rapid HIV antibody test**	r		NA	100% (98.2–100%)	NA	[36]
**Bacteriologic coverage rate**	rtx		80% (73–94%)	80% (73–94%)	80% (73–94%)	[37]
**Percentage of HIV+ individuals who are TST+**	tst		NA	70% (64.3–74.5%)	70% (64.3–74.5%)	[38]
**Effectiveness of ART in reversing effect of HIV on TB natural history**	$treat$		NA	NA	0.7 (0.47–0.87)	[39-41]
