# Supplementary material for: Integrating Community-Based Interventions to Reverse the Convergent TB/HIV Epidemics in Rural South Africa
Source: PLoS One. 2015 May 4;10(5):e0126267. doi: 10.1371/journal.pone.0126267 (PMC4418809; doi:10.1371/journal.pone.0126267)
Supplement: S2 Table — (PDF) [file pone.0126267.s006.pdf]

S2 Table. Additional Model Calibration and Validation.

Statistic	Model	Data	Year and Source
TB case detection rate	59.7%	67% (56% – 81%)	[2]
MDR-TB prevalence	4.25%	1.1% – 4.8%	[19]
Ratio XDR/MDR prevalence	12.0%	1.2% – 53.1%	[19]
ART coverage of eligible cases (CD4+<200 cells/ml per SA ART guidelines 2011 and earlier)	75.6%	79.0%	[27]
