# Supplementary material for: Integrating Community-Based Interventions to Reverse the Convergent TB/HIV Epidemics in Rural South Africa
Source: PLoS One. 2015 May 4;10(5):e0126267. doi: 10.1371/journal.pone.0126267 (PMC4418809; doi:10.1371/journal.pone.0126267)
Supplement: S3 Table — Partial rank correlation coefficients (PRCCs) and p-values calculated from model outcomes generated from minimum and maximum of parameter distributions found in S1 Table in a global sensitivity analysis of annual CICF screening. (PDF) [file pone.0126267.s007.pdf]

S3 Table. Global Sensitivity Analysis. Partial rank correlation coefficients (PRCCs) and p-values calculated from model outcomes generated from minimum and maximum of parameter distributions found in S1 Table in a global sensitivity analysis of annual CICF screening.

Parameters		***Total TB Cases Averted over 10 Years***		***DS-TB Cases Averted over 10 Years***		***MDR-TB Cases Averted over 10 Years***		***XDR-TB Cases Averted over 10 Years***		***HIV Infections Averted over 10 Years***		***TB/HIV Deaths Averted over 10 Years***	
		PRCC	*p-value*	*PRCC*	*p-value*	*PRCC*	*p-value*	*PRCC*	*p-value*	*PRCC*	*p-value*	*PRCC*	*p-value*
sm		0.025	0.450	0.024	0.468	-0.005	0.882	-0.007	0.830	0.011	0.743	0.028	0.398
σ		0.154	<0.01	0.027	0.405	0.312	<0.01	0.304	<0.01	0.025	0.435	0.028	0.397
sx	*HIV-*	0.456	<0.01	0.486	<0.01	0.354	<0.01	0.565	<0.01	-0.112	<0.01	0.176	<0.01
	HIV+/ART	0.350	<0.01	0.426	<0.01	0.163	<0.01	0.157	<0.01	0.027	0.411	0.257	<0.01
r		0.048	0.140	0.026	0.429	-0.004	0.903	-0.027	0.400	0.038	0.248	0.010	0.752
ϕ_{HIV}		-0.007	0.837	0.026	0.417	0.053	0.106	-0.102	<0.01	0.135	<0.01	0.057	0.079
tst		0.016	0.633	0.002	0.952	-0.011	0.741	0.034	0.301	-0.006	0.862	-0.017	0.599
xpt		0.045	0.166	0.038	0.238	0.014	0.670	-0.008	0.814	-0.050	0.128	0.048	0.145
sm_{XPTi}		0.027	0.415	0.009	0.791	-0.023	0.477	0.003	0.928	-0.065	0.048	0.027	0.407
sm_{XPTn}		0.019	0.567	0.040	0.224	-0.029	0.371	0.046	0.156	0.036	0.265	-0.008	0.810
rtx		-0.002	0.947	-0.020	0.538	0.028	0.393	-0.001	0.964	0.043	0.189	-0.031	0.343
fit_2		0.228	<0.01	0.086	<0.01	-0.106	<0.01	0.091	0.005	-0.034	0.296	0.013	0.687
fit_3		0.254	<0.01	0.042	0.200	0.010	0.764	0.133	<0.01	-0.028	0.394	0.072	0.026
p	*HIV-*	0.719	<0.01	0.936	<0.01	-0.459	<0.01	-0.747	<0.01	-0.217	<0.01	0.498	<0.01
	HIV+/ART	0.257	<0.01	0.483	<0.01	0.070	0.032	-0.106	<0.01	-0.141	<0.01	0.164	<0.01
x_{TB}		0.427	<0.01	0.677	<0.01	0.010	0.758	-0.027	0.406	-0.147	<0.01	0.215	<0.01
ν	*HIV-*	-0.009	0.775	0.018	0.574	-0.021	0.520	0.009	0.775	0.024	0.465	0.041	0.211
	HIV+/ART	-0.111	<0.01	-0.257	<0.01	-0.140	<0.01	-0.258	<0.01	0.066	0.043	0.269	<0.01
τ	*HIV-*	0.032	0.330	0.157	<0.01	-0.015	0.645	-0.073	0.026	0.016	0.629	-0.014	0.673
	HIV+/ART	-0.025	0.443	-0.079	0.015	0.009	0.787	0.024	0.462	0.018	0.589	0.004	0.907
$\frac{1}{\kappa}$		-0.010	0.763	0.051	0.118	0.550	<0.01	0.787	<0.01	0.114	<0.01	0.187	<0.01
ω_1		-0.137	<0.01	-0.028	0.389	-0.108	<0.01	0.015	0.653	-0.003	0.921	-0.035	0.283
ω_2		-0.033	0.314	0.022	0.493	0.046	0.160	-0.178	<0.01	0.016	0.623	-0.006	0.846
$\frac{1}{\rho_1}$	*DS-TB*	-0.025	0.436	-0.010	0.748	0.017	0.608	-0.017	0.600	0.028	0.392	0.032	0.329
$\frac{1}{\rho_2}$	*MDR-TB*	0.002	0.948	0.015	0.641	0.016	0.621	-0.001	0.972	0.048	0.139	0.014	0.663
$\frac{1}{\rho_3}$	*XDR-TB*	-0.035	0.282	-0.042	0.193	-0.007	0.828	-0.019	0.556	0.016	0.622	-0.033	0.313
q_{11}	*DS-TB*	0.101	<0.01	0.154	<0.01	0.025	0.448	-0.027	0.414	-0.017	0.600	-0.035	0.278
q_{12}, q_{22}	*MDR-TB*	0.141	<0.01	0.011	0.727	-	0.073	0.022	0.496	-0.062	0.055	0.006	0.851

						0.058							
q_{33}	*XDR-TB*	0.069	0.033	0.026	0.431	0.012	0.722	0.337	<0.01	-0.013	0.694	0.030	0.365
x_{HIV}		-0.104	<0.01	-0.118	<0.01	-0.067	0.040	-0.071	0.028	-0.789	<0.01	-0.105	<0.01
ϕ_{TB1}	*DS-TB*	0.183	<0.01	0.194	<0.01	-0.386	<0.01	-0.467	<0.01	-0.105	<0.01	0.055	0.093
$\phi_{TB2/3}$	*MDR- & XDR-TB*	-0.563	<0.01	0.084	0.010	-0.827	<0.01	-0.871	<0.01	0.014	0.659	-0.243	<0.01
d_{TB1}	*DS-TB*	0.041	0.211	-0.009	0.783	0.005	0.880	0.022	0.491	-0.016	0.633	0.032	0.321
$d_{TB2/3}$	*MDR- & XDR-TB*	0.002	0.959	-0.001	0.967	-0.025	0.434	0.018	0.588	0.001	0.985	0.002	0.959
d_{HIV}		-0.015	0.656	-0.040	0.217	0.013	0.690	0.021	0.515	0.032	0.320	0.052	0.111
$\frac{1}{\gamma}$	*TB-*	0.121	<0.01	0.121	<0.01	0.014	0.661	0.116	<0.01	0.694	<0.01	0.155	<0.01
	TB+	-0.040	0.219	0.028	0.397	-0.056	0.083	0.024	0.463	0.003	0.937	0.019	0.551
f	*HIV-*	0.097	<0.01	0.275	<0.01	-0.108	<0.01	-0.210	<0.01	-0.057	0.081	0.093	0.004
	HIV+/ART	0.341	<0.01	0.576	<0.01	-0.014	0.671	-0.285	<0.01	-0.118	<0.01	0.257	<0.01
ζ		-0.115	<0.01	-0.207	<0.01	-0.060	0.065	-0.065	0.045	0.009	0.787	-0.062	0.057
w		-0.018	0.591	0.071	0.029	-0.041	0.210	-0.038	0.239	0.000	0.991	-0.005	0.874
υ		-0.119	<0.01	-0.200	<0.01	-0.102	<0.01	-0.078	0.017	-0.012	0.710	0.001	0.979
β_{TB}		0.240	<0.01	0.525	<0.01	-0.138	<0.01	-0.280	<0.01	-0.073	0.024	0.117	<0.01
β_{HIV}		0.206	<0.01	0.286	<0.01	0.131	<0.01	0.013	0.700	-0.332	<0.01	0.445	<0.01
μ	*HIV-*	0.023	0.482	-0.039	0.227	-0.012	0.711	0.050	0.123	0.033	0.305	-0.023	0.481
	HIV+ or ART CD4 >350	-0.078	0.017	-0.163	<0.01	-0.012	0.717	0.190	<0.01	0.428	<0.01	-0.799	<0.01
	ART CD4 ≤350	0.054	0.095	0.032	0.332	0.054	0.098	0.089	<0.01	0.128	<0.01	-0.795	<0.01
	HIV+ CD4 ≤350	-0.602	<0.01	-0.693	<0.01	-0.441	<0.01	-0.395	<0.01	-0.906	<0.01	0.020	0.532
μ_{TB}	*HIV-*	-0.092	<0.01	-0.323	<0.01	-0.050	0.126	-0.026	0.430	0.010	0.765	0.023	0.487
	HIV+	-0.146	<0.01	-0.132	<0.01	-0.116	<0.01	-0.094	<0.01	-0.008	0.801	-0.046	0.158
	ART	0.139	<0.01	-0.146	<0.01	0.286	<0.01	0.529	<0.01	-0.006	0.862	0.007	0.822
δ		-0.432	<0.01	-0.548	<0.01	0.205	<0.01	0.299	<0.01	0.203	<0.01	-0.189	<0.01
η		-0.019	0.567	-0.023	0.474	-0.063	0.054	-0.049	0.136	-0.239	<0.01	-0.105	<0.01
vl		0.285	<0.01	0.363	<0.01	0.173	<0.01	0.123	<0.01	0.828	<0.01	0.443	<0.01
α		-0.241	<0.01	-0.263	<0.01	-0.085	<0.01	-0.016	0.620	0.344	<0.01	-0.433	<0.01
g		0.220	<0.01	0.453	<0.01	-0.001	0.969	-0.154	<0.01	0.036	0.269	0.253	<0.01
treat		0.490	<0.01	0.467	<0.01	0.492	<0.01	0.420	<0.01	0.004	0.905	0.056	0.088
