# Supplementary material for: Integrating Community-Based Interventions to Reverse the Convergent TB/HIV Epidemics in Rural South Africa
Source: PLoS One. 2015 May 4;10(5):e0126267. doi: 10.1371/journal.pone.0126267 (PMC4418809; doi:10.1371/journal.pone.0126267)
Supplement: S1 Text — Supplementary figures and tables are available as individual files, but are also included here for convenience, along with additional model details. (PDF) [file pone.0126267.s008.pdf]

Integrating Community-Based Interventions to Reverse the Convergent TB/HIV Epidemics in Rural South Africa

Technical Appendix

Jennifer A. Gilbert[1,2*], Elisa F. Long[3], Ralph P. Brooks[4], Gerald H. Friedland[1,4], Anthony P. Moll[4,5], Jeffrey P. Townsend[6,7,8], Alison P. Galvani[1,2,7,8], and Sheela V. Shenoi[4]

[1] Department of Epidemiology of Microbial Diseases, Yale School of Public Health, New Haven, CT, USA

[2] Center for Infectious Disease Modeling and Analysis, Yale School of Public Health, New Haven, CT, USA

[3] Anderson School of Management, University of California Los Angeles, Los Angeles, CA, USA

[4] Department of Medicine, Section of Infectious Diseases, AIDS Program, Yale University School of Medicine, New Haven, CT, USA

[5] Church of Scotland Hospital, Tugela Ferry, KwaZulu-Natal, South Africa

[6] Department of Biostatistics, Yale University, New Haven, CT, USA

[7] Department of Ecology and Evolutionary Biology, Yale University, New Haven, CT, USA

[8] Program in Computational Biology and Informatics, Yale University, New Haven, CT, USA

[*] Corresponding author

E-mail: jennifer.gilbert@yale.edu (JAG)

Model Calibration and Validation

We calibrated the model to TB and HIV longitudinal data from South Africa [1,2]. A small subset of parameters that could not be estimated from the literature or prior studies were fit to the data: population growth, TB and HIV transmission, background and disease-specific mortality, and treatment coverage parameters (S1 Table). For these parameters only, values were independently randomly sampled from plausible ranges derived from the literature, then supplied to model simulations to generate putative outcomes for TB and HIV incidence and prevalence. Outcomes were evaluated by least sums of squared differences to assess the goodness of fit of the predicted TB and HIV incidence and prevalence relative to the longitudinal data. Standard score normalization was used to weight all data points equally: $z = \frac{x-\mu}{\sigma}$, where z is the normalized data point, x is the data point before normalization, μ is the mean of the data points, and σ is the standard deviation of the data points. To identify the best-fit parameter values, we randomly sampled fitted parameter values to generate 100,000 model outcomes, selecting the set of parameters that produced the lowest sum of squared differences. Ranges for fitted parameters (used in sensitivity and uncertainty analyses) were determined by restricting parameter values to only those combinations that produced model outcome estimates within the minimum and maximum of the TB incidence, prevalence, and case detection rate longitudinal data.

To validate our model, we compared our model outcome predictions to the longitudinal data to which parameters were fit (S1 Fig. and S2 Table). We additionally compared our model predictions to observed data that were not used in the model calibration: prevalence of MDR and XDR-TB and estimated ART coverage (S2 Table).

Uncertainty and Sensitivity Analyses

To account for uncertainty in parameter estimates, we specified probability distributions encompassing values ranging from the minimum to maximum estimate for each parameter (S1 Table), and then evaluated the model outcomes predicted from random samples of those distributions. We specified Beta distributions for all parameters whose ranges were constrained between zero and one (*i.e.,* probabilities and proportions). Gamma distributions were specified for rate parameters. The means of the Beta and Gamma distributions were set to the best-fit parameter estimates. The variances were calculated based on the uniform distributions between the minimum and maximum estimates from the literature ($variance = \frac{1}{12}(maximum - minimum)^2$; S1 Table), and distributions were truncated by the minimum and maximum estimates. To efficiently draw a collection of variates from the probability distributions, Latin hypercube sampling was applied. These variates were used to parameterize the model and generate outcomes. The central 95% of each outcome was identified as representing our uncertainty. This uncertainty was demarcated by the error bars in the figures of the main results. Partial rank correlation coefficients (PRCCs) were calculated for model outcomes when CICF was implemented at an annual screening rate. PRCCs are listed in S3 Table.

Model Equations

Our model consists of differential equations, characterizing joint TB and HIV transmission in a rural community in South Africa. The modeled disease states are listed in S4 Table. For clarity, here we describe TB and HIV transmission separately, where X corresponds to the TB disease state; Y corresponds to the HIV disease state; i = infectious or noninfectious TB; and j = DS (j=1), MDR (j=2), or XDR (j=3) TB; ^{T} = TB; ^{H} = HIV.

Drug Susceptible TB

$$\frac{dS^TX^H}{dt} = g\left(1-\sum_j latent_j^{TB}\right)N+\psi P_S^TX^H-\left(\sum_j \lambda_j^{TB}+age\right)S^TX^H \tag{1}$$

$$\frac{dP_S^TX^H}{dt} = -\left(\sum_{j=2}^{3}\lambda_j^{TB}+\psi+age\right)P_S^TX^H \tag{2}$$

$$\begin{aligned}\frac{dL_1^TX^Hp_H}{dt} &= g*latent_1^{TB}*N+(1-p_H)\lambda_1^{TB}\left(S^TX^H+x_H^{TB}\sum_j\left(L_j^TX^H+E_j^TX^H+R_j^TX^H\right)\right) \\ &\quad +\zeta\left(A_{i,1}^TS^H+D_{i,1}^TS^H\right)+\psi P_{L1}^TX^H-\left(\nu_H+x_H^{TB}\sum_j\lambda_j^{TB}+age\right)L_1^TX^H\end{aligned} \tag{3}$$

$$\begin{aligned}\frac{dE_1^TX^H}{dt} &= p_H\lambda_1^{TB}\left(S^TX^H+x_H^{TB}\sum_j\left(L_j^TX^H+E_j^TX^H+R_j^TX^H\right)\right) \\ &\quad -\left(\tau_H+x_H^{TB}\sum_j\lambda_j^{TB}+age\right)E_1^TX^H\end{aligned} \tag{4}$$

$$\frac{dP_{L1}^TX^H}{dt} = -\left(x_H^{TB}\sum_{j=2}^{3}\lambda_j^{TB}+\psi+age\right)P_{L1}^TX^H \tag{5}$$

$$\begin{aligned}\frac{dA_{i1}^TX^H}{dt} &= f_H\left(\nu_HL_1^TX^H+\tau_HE_1^TX^H+\upsilon R_1^TX^H\right)+w_iA_{01}^TX^H+(1-\omega_1)\,d_1^{TB}\left(1-q_{11H}\right)\delta A_{i1}^TX^H \\ &\quad +\left((1-sx_H)+sx_H(1-sm_i)\,nocul+sx_H*sm_i*l_1^{TB}\right)\iota A_{i1}^TX^H \\ &\quad +sx_H*sm_i\left(1-l_1^{TB}\right)(1-\omega_1)\,d_1^{TB}\left(1-q_{11H}\right)\iota A_{i1}^TX^H \\ &\quad +\left((1-c_i)+l_1^{TB}c_i+\left(1-l_1^{TB}\right)(1-\omega_1)\,d_1^{TB}\left(1-q_{11H}\right)c_i\right)\sigma_{DS}D_1^TX^H \\ &\quad -\left(\delta+\iota+\zeta+\mu_{TB}+w_i+age\right)A_{i1}^TX^H\end{aligned} \tag{6}$$

$$\begin{aligned}\frac{dT_{i11}^TX^H}{dt} &= q_{11H}\delta A_{i1}^TX^H+q_{11H}\delta D_{i1}^TX^H+sx_H*sm_i\left(1-l_1^{TB}\right)q_{11H}\iota A_{i1}^TX^H+\left(1-l_1^{TB}\right)q_{11H}c_i\sigma_{DS}D_{i1}^TX^H \\ &\quad +q_{11H}\kappa F_{i11}^TX^H-\left(\rho_{DS}+age\right)T_{i11}^TX^H\end{aligned} \tag{7}$$

$$\begin{aligned}\frac{dF_{i11}^TX^H}{dt} &= (1-\omega_1)\left(1-d_1^{TB}\right)\left(\left(1-q_{11H}\right)\delta A_{i1}^TX^H+\left(1-q_{11H}\right)\delta D_{i1}^TX^H+sx_H*sm_i\left(1-l_1^{TB}\right)\left(1-q_{11H}\right)\iota A_{i1}^TX^H\right) \\ &\quad +(1-\omega_1)\left(1-d_1^{TB}\right)\left(1-l_1^{TB}\right)\left(1-q_{11H}\right)c_i\sigma_{DS}D_{i1}^TX^H+\left(1-q_{11H}\right)\kappa F_{i11}^TX^H \\ &\quad -\left(\mu_{TB}+\kappa+age\right)F_{i11}^TX^H\end{aligned} \tag{8}$$

$$\frac{dD_{i1}^TX^H}{dt} = sx_H(1-sm_i)\,cul\,\iota A_{i1}^TX^H+(1-\omega_1)\,d_1^{TB}\left(1-q_{11H}\right)\delta D_{i1}^TX^H-\left(\mu_{TB}+\sigma_{DS}+\delta+\zeta+age\right)D_{i1}^TX^H \tag{9}$$

$$\frac{dR_1^TX^H}{dt} = \rho_{DS}T_{i11}^TX^H-\left(\upsilon+x_H^{TB}\sum_j\lambda_j^{TB}+age\right)R_1^TX^H \tag{10}$$

$$\frac{dP_{R1}^TX^H}{dt} = -\left(x_H^{TB}\sum_{j=2}^{3}\lambda_j^{TB}+\psi+age\right)P_{R1}^TX^H \tag{11}$$

MDR-TB

$$\begin{aligned}
\frac{dL_2^T X^H}{dt} &= g * latent_2^{TB} * N \\
&+ (1 - p_H)\, \lambda_2^{TB} \left(S^T X^H + P_S^T X^H + x_H^{TB} \sum_j \left(L_j^T X^H + E_j^T X^H + R_j^T X^H + P_{Lj}^T X^H + P_{Rj}^T X^H \right) \right) \\
&+ \zeta \left(A_{i,2}^T S^H + D_{i,2}^T S^H \right) + \psi P_{L2}^T X^H - \left(\nu_H + x_H^{TB} \sum_j \lambda_j^{TB} + age \right) L_2^T X^H
\end{aligned} \tag{12}$$

$$\begin{aligned}
\frac{dE_2^T X^H}{dt} &= p_H \lambda_2^{TB} \left(S^T X^H + P_S^T X^H + x_H^{TB} \sum_j \left(L_j^T X^H + E_j^T X^H + R_j^T X^H + P_{Lj}^T X^H + P_{Rj}^T X^H \right) \right) \\
&- \left(\tau_H + x_H^{TB} \sum_j \lambda_j^{TB} + age \right) E_2^T X^H
\end{aligned} \tag{13}$$

$$\frac{dP_{L2}^T X^H}{dt} = - \left(\nu_H + x_H^{TB} \sum_{j=2}^{3} \lambda_j^{TB} + \psi + age \right) P_{L2}^T X^H \tag{14}$$

$$\frac{dA_{i2}^T X^H}{dt} = f_H \left(\nu_H \left(L_2^T X^H + P_{L2}^T X^H \right) + \tau_H E_2^T X^H + \upsilon \left(R_2^T X^H + P_{R2}^T X^H \right) \right) + w_i A_{02}^T X^H \tag{15}$$

$$\begin{aligned}
&+\omega_1 d_1^{TB} \left((1 - q_{11H})\, \delta A_{i1}^T X^H + sx_H * sm_i \left(1 - l_1^{TB} \right) (1 - q_{11H})\, \iota A_{i1}^T X^H + \left(1 - l_1^{TB} \right) (1 - q_{11H})\, c_i \sigma_{DS} D_{i1}^T X^H \right) \\
&+ \left((1 - rtx)\, d_1^{TB} (1 - q_{12H}) + rtx\, (1 - xpt)\, d_1^{TB} (1 - q_{12H}) + rtx * xpt\, (1 - \omega_2)\, d_2^{TB} (1 - q_{22H}) \right) \delta A_{i2}^T X^H \\
&\left((1 - sx_H) + sx_H (1 - sm_i)\, nocul + sx_H * sm_i\, (1 - xpt)\, nocul \left(l_1^{TB} + \left(1 - l_1^{TB} \right) d_1^{TB} (1 - q_{12H}) \right) \right) \iota A_{i2}^T X^H \\
&+ \left(sx_H * sm_i * xpt \left(l_2^{TB} + \left(1 - l_2^{TB} \right) (1 - \omega_2)\, d_2^{TB} (1 - q_{22H}) \right) \right) \iota A_{i2}^T X^H + (1 - c_i)\, \sigma_{DR} D_{i2}^T X^H \\
&+ l_2^{TB} \left(c_i \sigma_{DR} D_{i2}^T X^H + c_i \sigma_{DR} D_{Ti2}^T X^H \right) \\
&+ \left(1 - l_2^{TB} \right) (1 - \omega_2)\, d_2^{TB} \left(c_i (1 - q_{22H})\, \sigma_{DR} D_{i2}^T X^H + c_i (1 - q_{22H})\, \sigma_{DR} D_{Ti2}^T X^H \right) \\
&+ (1 - c_i)\, d_1^{TB} (1 - q_{12H})\, \sigma_{DR} D_{Ti2}^T X^H \\
&+ \left(xpt * l_2^{TB} + xpt \left(1 - l_2^{TB} \right) (1 - \omega_2)\, d_2^{TB} (1 - q_{22H}) \right) \kappa F_{i12}^T X^H + rtx * xpt * l_2^{TB} * \delta * A_{i2}^T X^H \qquad (16) \\
&- (\delta + \iota + \zeta + \mu_{TB} + w_i + age)\, A_{i2}^T X^H
\end{aligned}$$

$$\begin{aligned}
\frac{dT_{i12}^T X^H}{dt} &= (rtx\, (1 - xpt) + (1 - rtx))\, q_{12H} \delta A_{i2}^T X^H + sx_H * sm_i\, (1 - xpt)\, nocul \left(1 - l_1^{TB} \right) q_{12H} \iota A_{i2}^T X^H \\
&+ (1 - c_i)\, q_{12H} \sigma_{DR} D_{Ti2}^T X^H - (\rho_{DS} + age)\, T_{i12}^T X^H
\end{aligned} \tag{17}$$

$$\begin{aligned}
\frac{dT_{i22}^T X^H}{dt} &= rtx * xpt * q_{22H} \delta A_{i2}^T X^H + \left(1 - l_2^{TB} \right) q_{22H} c_i \sigma_{DR} D_{i2}^T X^H + \left(1 - l_2^{TB} \right) q_{22H} c_i \sigma_{DR} D_{Ti2}^T X^H \\
&+ xpt \left(1 - l_2^{TB} \right) q_{22H} \kappa F_{i12}^T X^H + sx_H * sm_i * xpt \left(1 - l_2^{TB} \right) q_{22H} \iota A_{i2}^T X^H + q_{22H} \kappa F_{i22}^T X^H \\
&- (\rho_{DR} + age)\, T_{i22}^T X^H
\end{aligned} \tag{18}$$

$$\begin{aligned}
\frac{dF_{i12}^T X^H}{dt} &= \omega_1 \left(1 - d_1^{TB} \right) \left((1 - q_{11H})\, \delta A_{i1}^T X^H + (1 - q_{11H})\, \delta D_{i1}^T X^H + sx_H * sm_i \left(1 - l_1^{TB} \right) (1 - q_{11H})\, \iota A_{i1}^T X^H \right) \\
&+ \omega_1 \left(1 - d_1^{TB} \right) \left(1 - l_1^{TB} \right) (1 - q_{11H})\, c_i \sigma_{DS} D_{i1}^T X^H + (rtx\, (1 - xpt) + (1 - rtx))\, (1 - q_{12H}) \left(1 - d_1^{TB} \right) \delta A_{i2}^T X^H \\
&+ sx_H * sm_i\, (1 - xpt)\, nocul \left(1 - l_1^{TB} \right) \left(1 - d_1^{TB} \right) (1 - q_{12H})\, \iota A_{i2}^T X^H \\
&+ (1 - c_i) \left(1 - d_1^{TB} \right) (1 - q_{12H})\, \sigma_{DR} D_{Ti2}^T X^H + (1 - xpt)\, nocul \kappa F_{i12}^T X^H \\
&- (\mu_{TB} + \kappa + age)\, F_{i12}^T X^H
\end{aligned} \tag{19}$$

$$\begin{aligned}
\frac{dF_{i22}^T X^H}{dt} &= rtx * xpt * (1 - \omega_2) \left(1 - d_2^{TB} \right) (1 - q_{22H})\, \delta A_{i2}^T X^H \\
&+ (1 - \omega_2) \left(1 - d_2^{TB} \right) \left(1 - l_2^{TB} \right) \left((1 - q_{22H})\, c_i \sigma_{DR} D_{i2}^T X^H + (1 - q_{22H})\, c_i \sigma_{DR} D_{Ti2}^T X^H + xpt\, (1 - q_{22H})\, \kappa F_{i12}^T X^H \right) \\
&+ sx_H * sm_i * xpt \left(1 - l_2^{TB} \right) (1 - \omega_2) \left(1 - d_2^{TB} \right) (1 - q_{22H})\, \iota A_{i2}^T X^H + (1 - q_{22H})\, \kappa F_{i22}^T X^H \\
&- (\mu_{TB} + \kappa + age)\, F_{i22}^T X^H
\end{aligned} \tag{20}$$

$$\begin{aligned}
\frac{dD_{i2}^T X^H}{dt} &= sx_H (1 - sm_i)\, cul \iota A_{i2}^T X^H + sx_H * sm_i\, (1 - xpt)\, cul * l_1^{TB} \iota A_{i2}^T X^H \\
&- (\mu_{TB} + \sigma_{DR} + \zeta + age)\, D_{i2}^T X^H
\end{aligned} \tag{21}$$

$$\begin{aligned}
\frac{dD_{Ti2}^T X^H}{dt} &= sx_H * sm_i \ (1 - xpt)\, cul \left(1 - l_2^{TB} \right) \iota A_{i2}^T X^H + (1 - xpt)\, cul \kappa F_{i12}^T X^H \\
&- (\mu_{TB} + \sigma_{DR} + age)\, D_{Ti2}^T X^H
\end{aligned} \tag{22}$$

$$\frac{dR_2^T X^H}{dt} = \rho_{DS} T_{i12}^T X^H + \rho_{DR} T_{i22}^T X^H + \psi P_{R2}^T X^H - \left(\upsilon + x_H^{TB} \sum_j \lambda_j^{TB} + age \right) R_2^T X^H \tag{23}$$

$$\frac{dP_{R2}^T X^H}{dt} = - \left(\upsilon + x_H^{TB} \sum_{j=2}^{3} \lambda_j^{TB} + \psi + age \right) P_{R2}^T X^H \tag{24}$$

XDR-TB

$$\begin{aligned}\frac{dL_3^T X^H}{dt} &= g * latent_3^{TB} * N \\ &+ (1-p_H)\lambda_3^{TB}\left(S^T X^H + P_S^T X^H + x_H^{TB}\sum_j \left(L_j^T X^H + E_j^T X^H + R_j^T X^H + P_{Lj}^T X^H + P_{Rj}^T X^H\right)\right) \\ &+\zeta\left(A_{i,3}^T S^H + D_{i,3}^T S^H\right) + \psi P_{L3}^T X^H - \left(\nu_H + x_H^{TB}\sum_j \lambda_j^{TB} + age\right) L_3^T X^H\end{aligned} \tag{25}$$

$$\begin{aligned}\frac{dE_3^T X^H}{dt} &= p_H \lambda_3^{TB}\left(S^T X^H + P_S^T X^H + x_H^{TB}\sum_j \left(L_j^T X^H + E_j^T X^H + R_j^T X^H + P_{Lj}^T X^H + P_{Rj}^T X^H\right)\right) \\ &- \left(\tau_H + x_H^{TB}\sum_j \lambda_j^{TB} + age\right) E_3^T X^H\end{aligned} \tag{26}$$

$$\frac{dP_{L3}^T X^H}{dt} = -\left(\nu_H + x_H^{TB}\sum_{j=2}^{3}\lambda_j^{TB} + \psi + age\right) P_{L3}^T X^H \tag{27}$$

$$\begin{aligned}\frac{dA_{i3}^T X^H}{dt} &= f_H\left(\nu_H\left(L_3^T X^H + P_{L3}^T X^H\right) + \tau_H E_3^T X^H + \upsilon\left(R_3^T X^H + P_{R3}^T X^H\right)\right) + w_i A_{03}^T X^H \\ &+rtx * xpt * \omega_2 d_2^{TB}\left(1-q_{22H}\right)\delta A_{i2}^T X^H \\ &+\omega_2 d_2^{TB}\left(1-l_2^{TB}\right)\left(\left(1-q_{22H}\right)c_i\sigma_{DR}D_{i2}^T X^H + \left(1-q_{22H}\right)c_i\sigma_{DR}D_{Ti2}^T X^H + xpt\left(1-q_{22H}\right)\kappa F_{i12}^T X^H\right) \\ &+sx_H * sm_i * xpt\left(1-l_2^{TB}\right)\omega_2 d_2^{TB}\left(1-q_{22H}\right)\iota A_{i2}^T X^H \\ &+\left(rtx * xpt * nocul\left(l_2^{TB} + \left(1-l_2^{TB}\right)d_2^{TB}\right) + rtx\left(1-xpt\right)\left(l_1^{TB} + \left(1-l_1^{TB}\right)d_1^{TB}\right) + \left(1-rtx\right)\left(l_1^{TB} + \left(1-l_1^{TB}\right)d_1^{TB}\right)\right)\delta A_{i3}^T X^H \\ &+\left(\left(1-sx_H\right) + sx_H\left(1-sm_i\right)nocul + sx_H * sm_i\left(1-xpt\right)nocul\left(l_1^{TB} + \left(1-l_1^{TB}\right)d_1^{TB}\right)\right)\iota A_{i3}^T X^H \\ &+\left(sx_H * sm_i * xpt * nocul\left(l_2^{TB} + \left(1-l_2^{TB}\right)d_2^{TB}\right)\right)\iota A_{i3}^T X^H \\ &+\left(1-l_3^{TB}\right)d_3^{TB}\left(\left(1-q_{33H}\right)c_i\sigma_{DR}D_{i3}^T X^H + \left(1-q_{33H}\right)c_i\sigma_{DR}D_{Ti13}^T X^H + \left(1-q_{33H}\right)c_i\sigma_{DR}D_{Ti23}^T X^H\right) \\ &+l_3^{TB}\left(c_i\sigma_{DR}D_{i3}^T X^H + c_i\sigma_{DR}D_{Ti13}^T X^H + c_i\sigma_{DR}D_{Ti23}^T X^H\right) \\ &+\left(1-c_i\right)\sigma_{DR}D_{i3}^T X^H + \left(1-c_i\right)d_1^{TB}\sigma_{DR}D_{Ti13}^T X^H + \left(1-c_i\right)d_1^{TB}\sigma_{DR}D_{Ti23}^T X^H + xpt * nocul\left(l_2^{TB} + \left(1-l_2^{TB}\right)d_2^{TB}\right)\kappa F_{i13}^T X^H \\ &+rtx * xpt * l_2^{TB}\delta A_{i3}^T X^H - \left(\delta + \iota + \zeta + \mu_{TB} + w_i + age\right)A_{i3}^T X^H\end{aligned} \tag{28}$$

$$\begin{aligned}\frac{dT_{i33}^T X^H}{dt} &= \left(1-l_3^{TB}\right)q_{33H}c_i\sigma_{DR}D_{i3}^T X^H + \left(1-l_3^{TB}\right)q_{33H}c_i\sigma_{DR}D_{Ti13}^T X^H + \left(1-l_3^{TB}\right)q_{33H}c_i\sigma_{DR}D_{Ti23}^T X^H + q_{33H}\kappa F_{i33}^T X^H \\ &-\left(\rho_{DR} + age\right)T_{i33}^T X^H\end{aligned} \tag{29}$$

$$\begin{aligned}\frac{dF_{i13}^T X^H}{dt} &= \left(rtx\left(1-xpt\right) + \left(1-rtx\right)\right)\left(1-l_1^{TB}\right)\left(1-d_1^{TB}\right)\delta A_{i3}^T X^H + sx_H * sm_i\left(1-xpt\right)nocul\left(1-l_1^{TB}\right)\left(1-d_1^{TB}\right)\iota A_{i3}^T X^H \\ &+\left(1-c_i\right)\left(1-d_1^{TB}\right)\sigma_{DR}D_{Ti13}^T X^H + \left(1-xpt\right)nocul\kappa F_{i13}^T X^H \\ &-\left(\mu_{TB} + \kappa + age\right)F_{i13}^T X^H\end{aligned} \tag{30}$$

$$\begin{aligned}\frac{dF_{i23}^T X^H}{dt} &= rtx * xpt * \omega_2\left(1-d_2^{TB}\right)\left(1-q_{22H}\right)\delta A_{i2}^T X^H \\ &+\left(1-l_2^{TB}\right)\left(1-d_2^{TB}\right)\omega_2\left(\left(1-q_{22H}\right)c_i\sigma_{DR}D_{i2}^T X^H + \left(1-q_{22H}\right)c_i\sigma_{DR}D_{Ti2}^T X^H + xpt\left(1-q_{22H}\right)\kappa F_{i12}^T\right) \\ &+sx_H * sm_i * xpt\left(1-l_2^{TB}\right)\omega_2\left(1-d_2^{TB}\right)\left(1-q_{22H}\right)\iota A_{i2}^T X^H + rtx * xpt * nocul\left(1-l_2^{TB}\right)\left(1-d_2^{TB}\right)\delta A_{i3}^T X^H \\ &+sx_H * sm_i * xpt * nocul\left(1-l_2^{TB}\right)\left(1-d_2^{TB}\right)\iota A_{i3}^T X^H + \left(1-c_i\right)\left(1-d_2^{TB}\right)\sigma_{DR}D_{Ti23}^T X^H \\ &+xpt * nocul\left(1-l_2^{TB}\right)\left(1-d_2^{TB}\right)\kappa F_{i13}^T X^H + nocul\kappa F_{i23}^T X^H \\ &-\left(\mu_{TB} + \kappa + age\right)F_{i23}^T X^H\end{aligned} \tag{31}$$

$$\begin{aligned}\frac{dF_{i33}^T X^H}{dt} &= \left(1-l_3^{TB}\right)\left(1-d_3^{TB}\right)\left(\left(1-q_{33H}\right)c_i\sigma_{DR}D_{i3}^T X^H + \left(1-q_{33H}\right)c_i\sigma_{DR}D_{Ti13}^T X^H + \left(1-q_{33H}\right)c_i\sigma_{DR}D_{Ti23}^T X^H\right) \\ &+\left(1-q_{33H}\right)\kappa F_{i33}^T X^H - \left(\mu_{TB} + \kappa + age\right)F_{i33}^T X^H\end{aligned} \tag{32}$$

$$\begin{aligned}\frac{dD_{i3}^T X^H}{dt} &= sx_H\left(1-sm_i\right)cul\iota A_{i3}^T X^H + sx_H * sm_i * xpt * cul * l_2^{TB}\iota A_{i3}^T X^H + sx_H * sm_i\left(1-xpt\right)cul * l_1^{TB}\iota A_{i3}^T X^H \\ &-\left(\mu_{TB} + \sigma_{DR} + \zeta + age\right)D_{i3}^T X^H\end{aligned} \tag{33}$$

$$\begin{aligned}\frac{dD_{Ti13}^T X^H}{dt} &= sx_H * sm_i\left(1-xpt\right)cul\left(1-l_1^{TB}\right)\iota A_{i3}^T X^H + \left(1-xpt\right)cul\kappa F_{i13}^T X^H \\ &-\left(\mu_{TB} + \sigma_{DR} + age\right)D_{Ti13}^T X^H\end{aligned} \tag{34}$$

$$\begin{aligned}\frac{dD_{Ti23}^T X^H}{dt} &= sx_H * sm_i * xpt * cul\left(1-l_2^{TB}\right)\iota A_{i3}^T X^H + rtx * xpt * cul * \left(1-l_2^{TB}\right)\delta A_{i3}^T X^H \\ &+cul\kappa F_{i23}^T X^H + xpt * cul\kappa F_{i13}^T X^H - \left(\mu_{TB} + \sigma_{DR} + age\right)D_{Ti23}^T X^H\end{aligned} \tag{35}$$

$$\frac{dR_3^T X^H}{dt} = \rho_{DR}T_{i33}^T X^H + \psi P_{R3}^T X^H - \left(\upsilon + x_H^{TB}\sum_j \lambda_j^{TB} + age\right)R_3^T X^H \tag{36}$$

$$\frac{dP_{R3}^T X^H}{dt} = -\left(\upsilon + x_H^{TB}\sum_{j=2}^{3}\lambda_j^{TB} + \psi + age\right)P_{R3}^T X^H \tag{37}$$

HIV

$$\frac{dY^T S^H}{dt} = -\left(\mu_H + \lambda_{HIV}\right) Y^T S^H \tag{38}$$

$$\frac{dY^T I^H}{dt} = \lambda_{HIV} Y^T S^H + \left(l_{HIV} * r + (1-r)\right) \epsilon Y^T I^H + d_{HIV} Y^T I^H_{ART} - \left(\mu_H + \gamma + \eta + \epsilon\right) Y^T I^H \tag{39}$$

$$\frac{dY^T E^H}{dt} = \gamma Y^T I^H + \left(l_{HIV} * r + (1-r)\right) \epsilon Y^T E^H + d_{HIV} Y^T E^H_{ART} \left(\mu_H + \eta + \epsilon\right) Y^T E^H \tag{40}$$

$$\frac{dY^T I^H_{ART}}{dt} = \eta Y^T I^H + \left(1 - l_{HIV}\right) r\epsilon Y^T I^H - \left(\mu_H + d_{HIV}\right) Y^T I^H_{ART} \tag{41}$$

$$\frac{dY^T E^H_{ART}}{dt} = \eta Y^T E^H + \left(1 - l_{HIV}\right) r\epsilon Y^T E^H - \left(\mu_H + d_{HIV}\right) Y^T E^H_{ART} \tag{42}$$

Other

$$\lambda_j^{TB} = \beta_{TB} \left(1 - fit_j\right) \left(A^T_{ij} X^H + F^T_{ij} X^H + D^T_{ij} X^H + D^T_{Tij} X^H\right) \tag{43}$$

$$\lambda^{HIV} = \beta_{HIV} e^{\left(-\alpha \frac{Y^T I^H + Y^T E^H + Y^T I^H_{ART} + Y^T E^H_{ART}}{N}\right)} \left(Y^T I^H + vl Y^T E^H + x_{HIV} Y^T I^H_{ART} + vl * x_{HIV} Y^T E^H_{ART}\right) \tag{44}$$

References

1. Actuarial Society of South Africa (2011) ASSA2008 Model.
2. WHO (2014) WHO TB Burden Estimates.
3. Basu S, Andrews JR, Poolman EM, Gandhi NR, Shah NS, et al. (2007) Prevention of nosocomial transmission of extensively drug-resistant tuberculosis in rural South African district hospitals: an epidemiological modelling study. Lancet 370: 1500-1507.
4. Dowdy DW, Chaisson RE, Maartens G, Corbett EL, Dorman SE (2008) Impact of enhanced tuberculosis diagnosis in South Africa: a mathematical model of expanded culture and drug susceptibility testing. Proc Natl Acad Sci U S A 105: 11293-11298.
5. Martin DJ, Sim JG, Sole GJ, Rymer L, Shalekoff S, et al. (1995) CD4+ lymphocyte count in African patients co-infected with HIV and tuberculosis. J Acquir Immune Defic Syndr Hum Retrovirol 8: 386-391.
6. Lawn SD, Harries AD, Williams BG, Chaisson RE, Losina E, et al. (2011) Antiretroviral therapy and the control of HIV-associated tuberculosis. Will ART do it? Int J Tuberc Lung Dis 15: 571-581.
7. Havlir DV, Getahun H, Sanne I, Nunn P (2008) Opportunities and challenges for HIV care in overlapping HIV and TB epidemics. JAMA 300: 423-430.
8. Modjarrad K, Vermund SH (2010) Effect of treating co-infections on HIV-1 viral load: a systematic review. Lancet Infect Dis 10: 455-463.
9. Cohen T, Murray M (2004) Modeling epidemics of multidrug-resistant M. tuberculosis of heterogeneous fitness. Nat Med 10: 1117-1121.
10. Basu S, Friedland GH, Medlock J, Andrews JR, Shah NS, et al. (2009) Averting epidemics of extensively drug-resistant tuberculosis. Proc Natl Acad Sci U S A 106: 7672-7677.
11. Dye C, Garnett GP, Sleeman K, Williams BG (1998) Prospects for worldwide tuberculosis control under the WHO DOTS strategy. Directly observed short-course therapy. Lancet 352: 1886-1891.
12. Gandhi NR, Moll A, Sturm AW, Pawinski R, Govender T, et al. (2006) Extensively drug-resistant tuberculosis as a cause of death in patients co-infected with tuberculosis and HIV in a rural area of South Africa. Lancet 368: 1575-1580.
13. South Africa Department of Health (2006) The KZN "outbreak". Pretoria: DoH.
14. Orenstein EW, Basu S, Shah NS, Andrews JR, Friedland GH, et al. (2009) Treatment outcomes among patients with multidrug-resistant tuberculosis: systematic review and meta-analysis. Lancet Infect Dis 9: 153-161.
15. Jacobson KR, Tierney DB, Jeon CY, Mitnick CD, Murray MB (2010) Treatment outcomes among patients with extensively drug-resistant tuberculosis: systematic review and meta-analysis. Clin Infect Dis 51: 6-14.
16. WHO (2010) Treatment of Tuberculosis Guidelines. Fourth edition. Geneva, Switzerlnd: WHO.
17. Republic of South Africa (2011) Multi-Drug Resistant Tuberculosis. A Policy Framework on Decentralised and Deinstitutionalised Management for South Africa.
18. Health Systems Trust (2013) Defaulter (interruption) rate (new Sm+ cases) (%).
19. Wallengren K, Scano F, Nunn P, Margot B, Buthelezi SS, et al. (2011) Drug-Resistant tuberculosis, KwaZulu-Natal, South Africa, 2001-2007. Emerg Infect Dis 17: 1913-1916.

20. Holtz TH, Lancaster J, Laserson KF, Wells CD, Thorpe L, et al. (2006) Risk factors associated with default from multidrug-resistant tuberculosis treatment, South Africa, 1999-2001. Int J Tuberc Lung Dis 10: 649-655.
21. Shean KP, Willcox PA, Siwendu SN, Laserson KF, Gross L, et al. (2008) Treatment outcome and follow-up of multidrug-resistant tuberculosis patients, West Coast/Winelands, South Africa, 1992-2002. Int J Tuberc Lung Dis 12: 1182-1189.
22. Mlambo CK, Warren RM, Poswa X, Victor TC, Duse AG, et al. (2008) Genotypic diversity of extensively drug-resistant tuberculosis (XDR-TB) in South Africa. Int J Tuberc Lung Dis 12: 99-104.
23. Loveday M, Wallengren K, Voce A, Margot B, Reddy T, et al. (2012) Comparing early treatment outcomes of MDR-TB in decentralised and centralised settings in KwaZulu-Natal, South Africa. Int J Tuberc Lung Dis 16: 209-215.
24. Dowdy DW, O'Brien MA, Bishai D (2008) Cost-effectiveness of novel diagnostic tools for the diagnosis of tuberculosis. Int J Tuberc Lung Dis 12: 1021-1029.
25. Gandhi NR, Moll AP, Lalloo U, Pawinski R, Zeller K, et al. (2009) Successful integration of tuberculosis and HIV treatment in rural South Africa: the Sizonq'oba study. J Acquir Immune Defic Syndr 50: 37-43.
26. Long EF, Brandeau ML, Owens DK (2010) The cost-effectiveness and population outcomes of expanded HIV screening and antiretroviral treatment in the United States. Ann Intern Med 153: 778-789.
27. Johnson LF (2012) Access to Antiretroviral Treatment in South Africa, 2004-2011. The Southern African Journal of HIV Medicine 13: 22-27.
28. Bassett IV, Regan S, Chetty S, Giddy J, Uhler LM, et al. (2010) Who starts antiretroviral therapy in Durban, South Africa?... not everyone who should. AIDS 24 Suppl 1: S37-44.
29. Rosen S, Fox MP (2011) Retention in HIV care between testing and treatment in sub-Saharan Africa: a systematic review. PLoS Med 8: e1001056.
30. Fox MP, Rosen S (2010) Patient retention in antiretroviral therapy programs up to three years on treatment in sub-Saharan Africa, 2007-2009: systematic review. Trop Med Int Health 15 Suppl 1: 1-15.
31. Ayles H, Schaap A, Nota A, Sismanidis C, Tembwe R, et al. (2009) Prevalence of tuberculosis, HIV and respiratory symptoms in two Zambian communities: implications for tuberculosis control in the era of HIV. PLoS One 4: e5602.
32. Getahun H, Kittikraisak W, Heilig CM, Corbett EL, Ayles H, et al. (2011) Development of a standardized screening rule for tuberculosis in people living with HIV in resource-constrained settings: individual participant data meta-analysis of observational studies. PLoS Med 8: e1000391.
33. Hernandez-Garduno E, Cook V, Kunimoto D, Elwood RK, Black WA, et al. (2004) Transmission of tuberculosis from smear negative patients: a molecular epidemiology study. Thorax 59: 286-290.
34. Steingart KR, Ng V, Henry M, Hopewell PC, Ramsay A, et al. (2006) Sputum processing methods to improve the sensitivity of smear microscopy for tuberculosis: a systematic review. Lancet Infect Dis 6: 664-674.
35. Boehme C, Nicol M, Nabeta P, Michael J, Gotuzzo E, et al. (2011) Feasibility, diagnostic accuracy, and effectiveness of decentralised use of the Xpert MTB/RIF test for diagnosis of tuberculosis and multidrug resistance: a multicentre implementation study. Lancet 377: 1495-1505.

36. Branson DM (2003) Point-of-Care Rapid Tests for HIV Antibodies. Journal of Laboratory Medicine 27: 288-295.
37. Health Systems Trust (2013) Bacteriological coverage rate (%.
38. Cobelens FG, Egwaga SM, van Ginkel T, Muwinge H, Matee MI, et al. (2006) Tuberculin skin testing in patients with HIV infection: limited benefit of reduced cutoff values. Clin Infect Dis 43: 634-639.
39. Badri M, Wilson D, Wood R (2002) Effect of highly active antiretroviral therapy on incidence of tuberculosis in South Africa: a cohort study. Lancet 359: 2059-2064.
40. Golub JE, Saraceni V, Cavalcante SC, Pacheco AG, Moulton LH, et al. (2007) The impact of antiretroviral therapy and isoniazid preventive therapy on tuberculosis incidence in HIV-infected patients in Rio de Janeiro, Brazil. AIDS 21: 1441-1448.
41. Lawn SD, Kranzer K, Wood R (2009) Antiretroviral therapy for control of the HIV-associated tuberculosis epidemic in resource-limited settings. Clin Chest Med 30: 685-699, viii.

S1 Fig. Model Calibration and Validation. Model calibration and validation using (A) TB incidence, (B) TB prevalence, and (C) HIV prevalence data. Error bars indicate available minimum and maximum values in data.

A

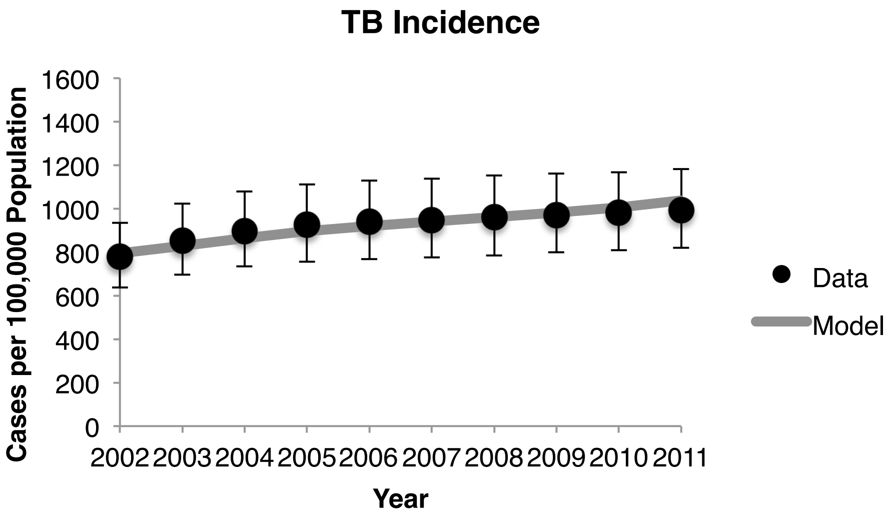

B

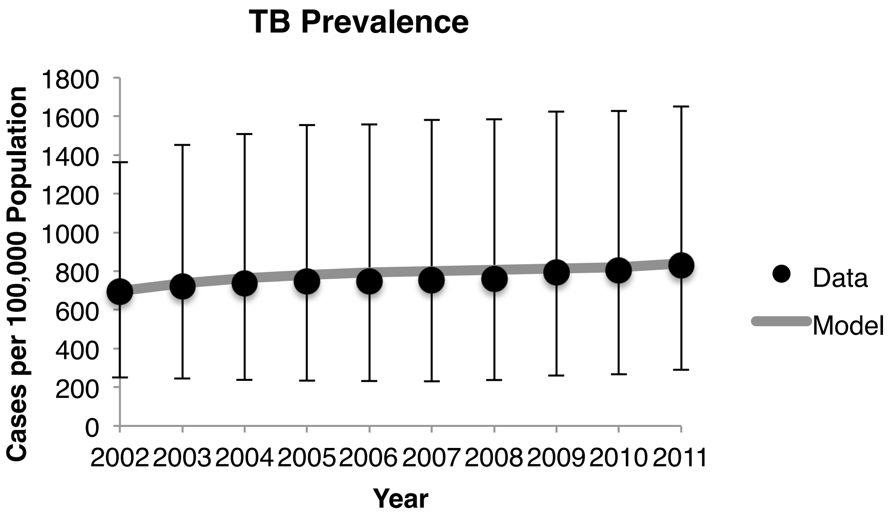

C

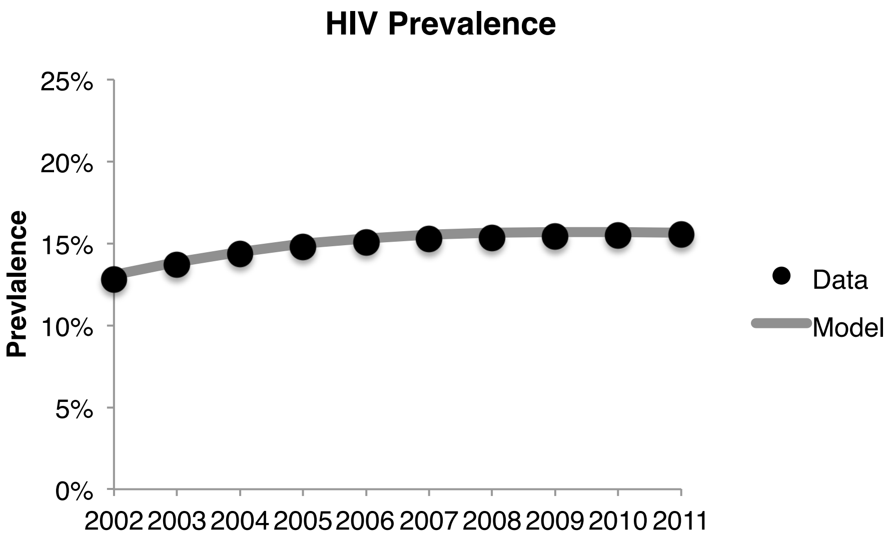
HIV Prevalence
Prevlalence
25%
20%
15%
10%
5%
0%
2002 2003 2004 2005 2006 2007 2008 2009 2010 2011
Data
Model

S2 Fig. CICF with ART eligibility at CD4+ cell count ≤ 500 cells per milliliter. Impact of increasing eligibility to initiate ART from CD4+ cell count ≤ 350 cells per milliliter to a CD4+ cell count ≤ 500 cells per milliliter, and assuming ART coverage for individuals with a CD4+ cell count between 350 and 500 cells per milliliter is equal to the ART coverage for individuals with a CD4+ cell count below 350 cells per milliliter, on total TB cases averted, HIV infections averted, drug susceptible TB (DS TB) cases averted, MDR-TB cases averted, XDR-TB cases averted, and TB/HIV deaths averted by CICF at frequencies corresponding to screening individuals on average once every five years (annual frequency = 0.20), once every four years (annual frequency = 0.25), once every three years (annual frequency = 0.33), once every two years (annual frequency = 0.5), annually (annual frequency = 1), two times annually (annual frequency = 2), three times annually (annual frequency = 3), four times annually (annual frequency = 4), and five times annually (annual frequency = 5).

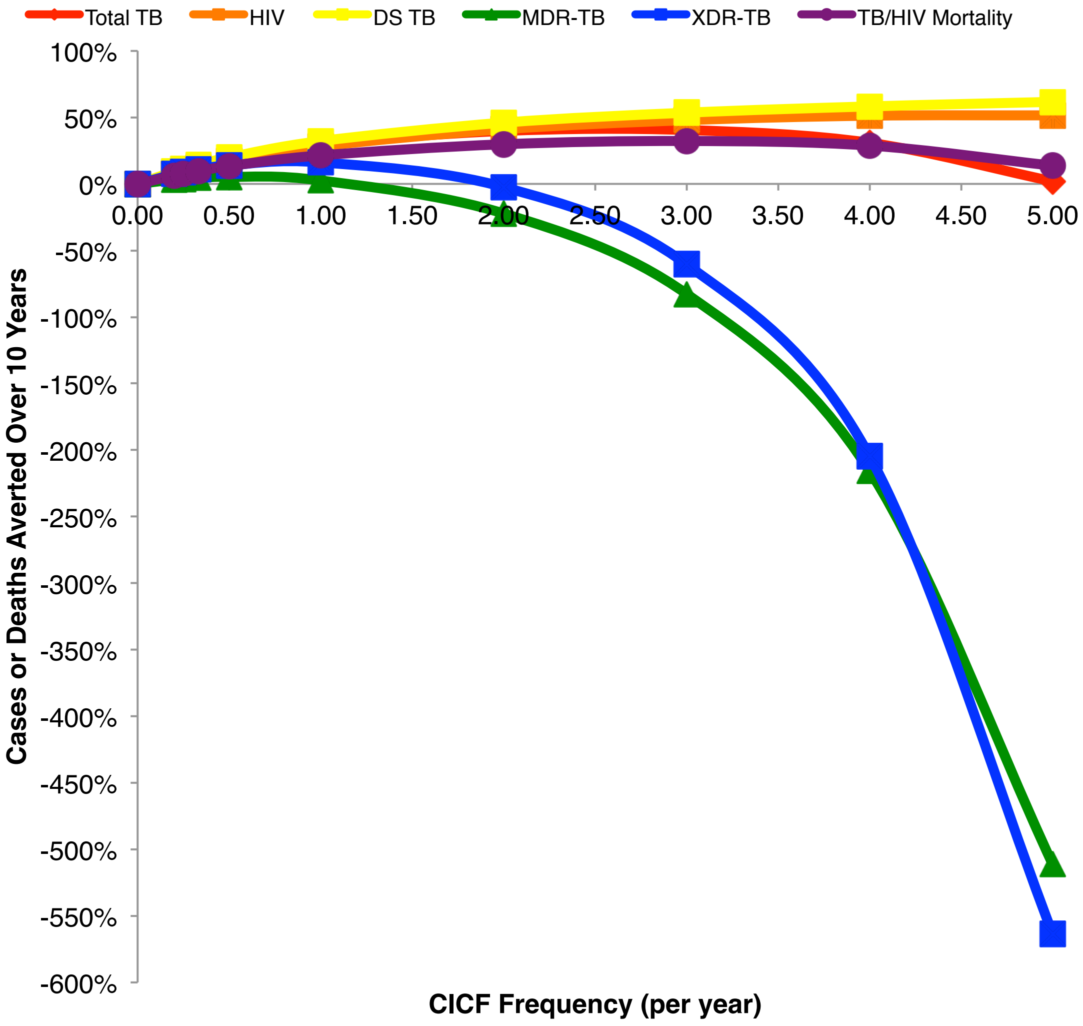

S3 Fig. Decreased ART Efficacy. Impact of decreasing ART efficacy on total TB cases averted, HIV infections averted, drug susceptible TB (DS TB) cases averted, MDR-TB cases averted, XDR-TB cases averted, and TB/HIV deaths averted by expanding ART coverage to 80% and increasing five-year retention to 70% by 2016. Note that Total TB and DS TB lines overlap because they are very close in value; the majority of total TB cases are drug susceptible.

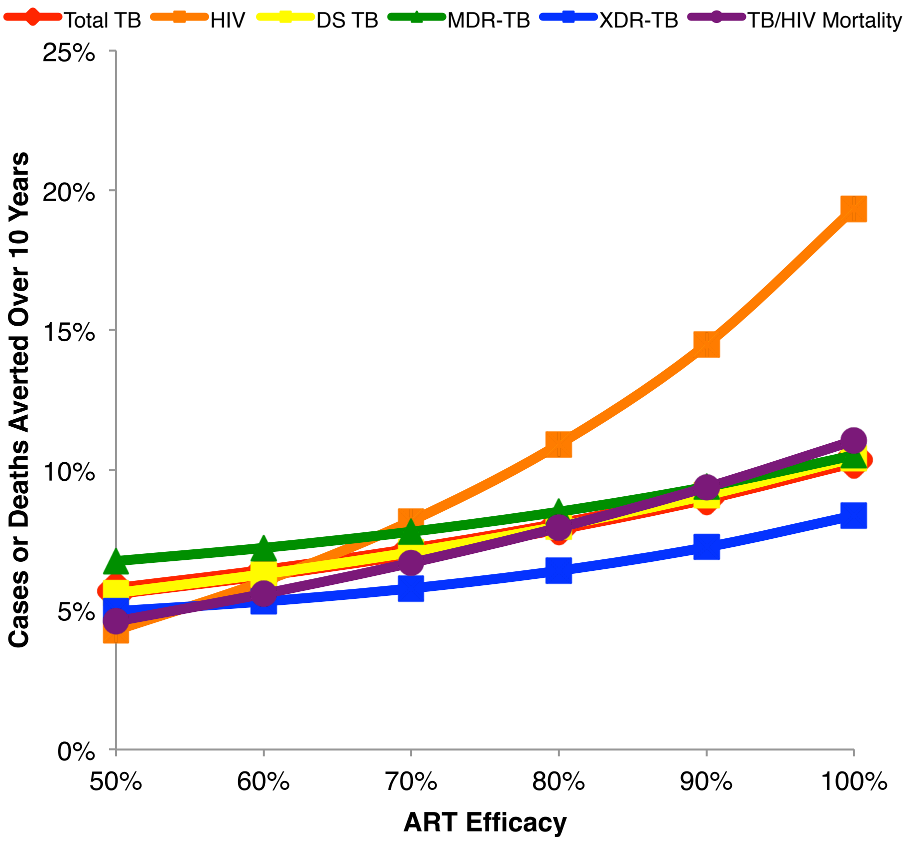

S4 Fig. Expansion of ART coverage with ART eligibility at CD4+ cell count ≤ 500 cells per milliliter. Impact of increasing eligibility to initiate ART from CD4+ cell count ≤ 350 cells per milliliter to a CD4+ cell count ≤ 500 cells per milliliter, with ART coverage for individuals with a CD4+ cell count between 350 and 500 cells per milliliter varied between 0% and 100% of the ART coverage for individuals with a CD4+ cell count ≤ 350 cells per milliliter, on total TB cases averted, HIV infections averted, drug susceptible TB (DS TB) cases averted, MDR-TB cases averted, XDR-TB cases averted, and TB/HIV deaths averted by expanding ART coverage to 80% and increasing five-year retention to 70% by 2016. Note that Total TB and DS TB lines overlap because they are very close in value; the majority of total TB cases are drug susceptible.

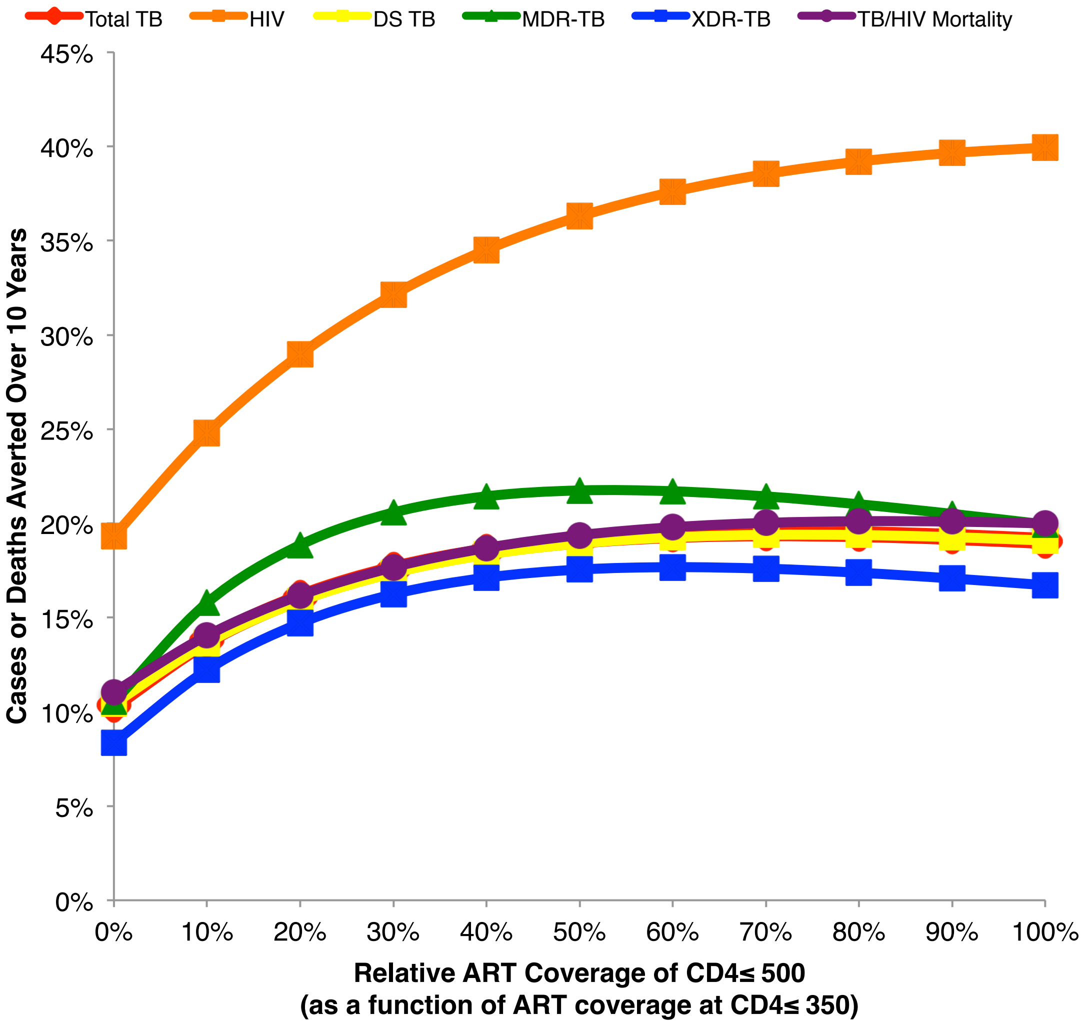

S1 Table. Model parameters, ranges, and references. Rates are in units of year^{-1} unless otherwise noted. *HIV+ individuals on ART have the effect of HIV on the natural history of TB reduced by the effectiveness of ART (parameter *treat*).

Parameter	Symbol	TB Disease Status	HIV–	HIV+ (No ART)	HIV+ (ART)	Reference
**Annual population growth rate**	g		0.0572 (0.02–0.06)	NA	NA	Fitted to [1,2]
**Annual mortality rate**	μ	*Baseline (no TB)*	0.003 (0.0009–0.004)	0.0155 (0.004–0.13, CD4+ >350), 0.339 (0.21–0.64, CD4+ ≤350)	0.0155 (0.004–0.13, CD4+ >350), 0.122 (0.004–0.13, CD4+ ≤350)	Fitted to [1,2], ranges adapted from [3-8]
	μ_{TB}	*Active TB*	0.30 (0.2–0.4)	0.992 (0.75–1)	0.508 (0.2–0.76)	Fitted to [1,2], ranges adapted from [3]
**TB transmissibility coefficient**	β_{TB}		9.52 x 10^{-5}	9.52 x 10^{-5}	9.52 x 10^{-5}	Fitted to [1,2]
**Relative fitness penalty drug resistance TB (proportion)**	fit_2	*MDR*	0.12 (0–0.7)	0.12 (0–0.7)	0.12 (0–0.7)	[3,9]
	fit_3	*XDR*	0.16 (0–0.7)	0.16 (–-0.7)	0.16 (0–0.7)	[3,9]
**Proportion who develop primary progressive TB**	p		0.14 (0.08–0.25)	0.67 (0.36–0.8)	*	[3,10]
**Endogenous annual TB reactivation rate**	ν	*Long latency*	0.00011 (0.0001–0.0003)	0.17 (0.04–0.2)	*	[3,10]
	τ	*Short latency*	0.88 (0.76–0.99)	12 (10.4–13.5)	*	[3,10]
**Degree susceptibility despite prior TB infection (proportion)**	x_{TB}		0.35 (0.1–0.6)	0.75 (0.5–1)	*	[3]
**Proportion of TB infections that are infectious**	f		0.65 (0.5–0.65)	0.3 (0.19–0.4)	*	[3]
**Rate of conversion from non-infectious to infectious TB**	w		0.015 (0.007–0.02)	0.015 (0.007–0.02)	0.015 (0.007–0.02)	[3,11]
**Rate of natural self cure**	ξ		0.2 (0.15–0.25)	0.2 (0.15–0.25)	0.2 (0.15–0.25)	[3]
**Baseline TB detection and treatment**	δ		1.0299 (0.8415–1.1137)	1.0299 (0.8415–1.1137)	1.0299 (0.8415–1.1137)	Fitted to [1,2]
**Proportion cured by 1st line TB drugs**	q_{11}	*DS*	0.73 (0.65–0.88)	0.58 (0.52–0.74)	*	[3,9]
	q_{12}	*MDR*	0.47 (0.18–0.58)	0.30 (0.16–0.36)	*	[3,9,12,13]
	q_{13}	*XDR*	0	0	0	[3,9,12,13]
**Proportion cured by 2nd line TB drugs**	q_{22}	*MDR*	0.67 (0.43–0.80)	0.45 (0.43–0.51)	*	[3,9,12-14]
	q_{33}	*XDR*	0.54 (0.328–0.545)	0.36 (0.328–0.400)	*	[3,12,15]
**Duration TB treatment (months)**	$\frac{1}{\rho_1}$	*DS*	6 (6–8)	6 (6–8)	6 (6–8)	[16]
	$\frac{1}{\rho_2}$	*MDR*	24 (18–24)	24 (18–24)	24 (18–24)	[17]
	$\frac{1}{\rho_3}$	*XDR*	26 (24.6–27.8)	26 (24.6–27.8)	26 (24.6–27.8)	[10]
**Proportion defaulting from TB treatment**	d_{TB1}	*DS*	0.07 (0.048–0.088)	0.07 (0.048–0.088)	0.07 (0.048–0.088)	[18]

	d_{TB2}	*MDR*	0.2 (0.19–0.37)	0.2 (0.19–0.37)	0.2 (0.19–0.37)	[19-21]
	d_{TB3}	*XDR*	0.28 (0.19–0.37)	0.28 (0.19–0.37)	0.28 (0.19–0.37)	[17,19,22]
**Proportion of treated TB patients who acquire or amplify resistance**	ω_1	*DS to MDR*	0.038 (0.025–0.1)	0.038 (0.025–0.1)	0.038 (0.025–0.1)	[3]
	ω_2	*MDR to XDR*	0.030 (0.025–0.1)	0.030 (0.025–0.1)	0.030 (0.025–0.1)	[3]
**Time to identification of TB treatment failure (months)**	$\frac{1}{\kappa}$		2 (1–4)	2 (1–4)	2 (1–4)	[10]
**Time from culture collection to MDR treatment initiation (days)**	$\frac{1}{\sigma_{DR}}$	*Baseline*	93 (71–120)	93 (71–120)	93 (71–120)	[23]
	$\frac{1}{\sigma_{DS}}$	*Decentralized*	72 (56–99)	72 (56–99)	72 (56–99)	[23]
**Proportion of cases defaulting before TB treatment initiation**	ϕ_{TB}	*DS outpatient (DOTS)*	0.15 (0–0.24)	0.15 (0–0.24)	0.15 (0–0.24)	[24]
		MDR inpatient (baseline)	0.5 (0.29–0.73)	0.5 (0.29–0.73)	0.5 (0.29–0.73)	[17]
		XDR inpatient	0.5 (0.29–0.73)	0.5 (0.29–0.73)	0.5 (0.29–0.73)	[17]
**HIV transmissibility coefficient**	β_{HIV}		NA	1.63×10^{-6}	1.63×10^{-6}	Fitted to [1,2]
**α (HIV transmission shape parameter)**	α		NA	4.55 (1–6)	4.55 (1–6)	Fitted to [1,2]
**Relative infectivity of HIV on ART (proportion)**	x_{HIV}		NA	NA	0.12 (0–0.12)	[25]
**Relative infectivity of ART eligible**	vl		NA	6.87 (1.00–3.33)	NA	Fitted to [1,2], Range from [26]
**ART coverage (CD4+ < 350)**	η		NA	50% (46–57%)	NA	[27]
**Proportion of cases detected defaulting before HIV treatment initiation**	ϕ_{HIV}		NA	0.38 (0.16–0.61)	NA	[28,29]
**Proportion retained in HIV treatment over 3 years**	d_{HIV}		NA	NA	0.75 (0.64–0.87)	[30]
**Rate of relapse from chemotherapeutic cure**	υ		0.001 (0–0.01)	0.001 (0–0.01)	0.001 (0–0.01)	[3]
**Duration of HIV infection, CD4+ cell count >350 (years)**	$\frac{1}{\gamma}$		NA	6 (5–7); 3 (2–4, active TB infx)	NA	[5-8]
**Sensitivity of TB symptom screening**	sx		69% (51.9–83.7%)	79% (58.3–90.9%)	79% (58.3–90.9%)	[31,32]
**Sensitivity of TB sputum smear**	sm_i	*Infectious*	83.3% (80–83.3%)	83.3% (80–83.3%)	83.3% (80–83.3%)	[3,33,34]
	sm_n	*Noninfectious*	0%	0%	0%	[3,33,34]
**Sensitivity of TB sputum culture/DST**	c_i	*Infectious*	100%	100%	100%	[4]
	c_n	*Noninfectious*	68%	68%	68%	[4]
**Sensitivity of Xpert MTB/RIF for TB diagnosis**	sm_{XPTi}	*Infectious*	98.3% (97–99%)	98.3% (97–99%)	98.3% (97–99%)	[35]
	sm_{XPTn}	*Noninfectious*	76.9% (72.4–80.8%)	76.9% (72.4–80.8%)	76.9% (72.4–80.8%)	[35]
**Sensitivity of Xpert MTB/RIF for RIF resistance detection given MTB detected**	xpt		94.4% (90.8–98.6%)	94.4% (90.8–98.6%)	94.4% (90.8–98.6%)	[35]
**Sensitivity of rapid HIV antibody test**	r		NA	100% (98.2–100%)	NA	[36]
**Bacteriologic coverage rate**	rtx		80% (73–94%)	80% (73–94%)	80% (73–94%)	[37]
**Percentage of HIV+ individuals who are**	tst		NA	70% (64.3–74.5%)	70% (64.3–74.5%)	[38]

**TST+**					
**Effectiveness of ART in reversing effect of HIV on TB natural history**	*treat*	NA	NA	0.7 (0.47–0.87)	[39-41]

S2 Table. Additional Model Calibration and Validation.

Statistic	Model	Data	Year and Source
TB case detection rate	59.7%	67% (56% – 81%)	[2]
MDR-TB prevalence	4.25%	1.1% – 4.8%	[19]
Ratio XDR/MDR prevalence	12.0%	1.2% – 53.1%	[19]
ART coverage of eligible cases (CD4+<200 cells/ml per SA ART guidelines 2011 and earlier)	75.6%	79.0%	[27]

S3 Table. Global Sensitivity Analysis. Partial rank correlation coefficients (PRCCs) and p-values calculated from model outcomes generated from minimum and maximum of parameter distributions found in S1 Table in a global sensitivity analysis of annual CICF screening.

Parameters		***Total TB Cases Averted over 10 Years***		***DS-TB Cases Averted over 10 Years***		***MDR-TB Cases Averted over 10 Years***		***XDR-TB Cases Averted over 10 Years***		***HIV Infections Averted over 10 Years***		***TB/HIV Deaths Averted over 10 Years***	
		PRCC	*p-value*	*PRCC*	*p-value*	*PRCC*	*p-value*	*PRCC*	*p-value*	*PRCC*	*p-value*	*PRCC*	*p-value*
sm		0.025	0.450	0.024	0.468	-0.005	0.882	-0.007	0.830	0.011	0.743	0.028	0.398
σ		0.154	<0.01	0.027	0.405	0.312	<0.01	0.304	<0.01	0.025	0.435	0.028	0.397
sx	*HIV-*	0.456	<0.01	0.486	<0.01	0.354	<0.01	0.565	<0.01	-0.112	<0.01	0.176	<0.01
	HIV+/ART	0.350	<0.01	0.426	<0.01	0.163	<0.01	0.157	<0.01	0.027	0.411	0.257	<0.01
r		0.048	0.140	0.026	0.429	-0.004	0.903	-0.027	0.400	0.038	0.248	0.010	0.752
ϕ_{HIV}		-0.007	0.837	0.026	0.417	0.053	0.106	-0.102	<0.01	0.135	<0.01	0.057	0.079
tst		0.016	0.633	0.002	0.952	-0.011	0.741	0.034	0.301	-0.006	0.862	-0.017	0.599
xpt		0.045	0.166	0.038	0.238	0.014	0.670	-0.008	0.814	-0.050	0.128	0.048	0.145
sm_{XPTi}		0.027	0.415	0.009	0.791	-0.023	0.477	0.003	0.928	-0.065	0.048	0.027	0.407
sm_{XPTn}		0.019	0.567	0.040	0.224	-0.029	0.371	0.046	0.156	0.036	0.265	-0.008	0.810
rtx		-0.002	0.947	-0.020	0.538	0.028	0.393	-0.001	0.964	0.043	0.189	-0.031	0.343
fit_2		0.228	<0.01	0.086	<0.01	-0.106	<0.01	0.091	0.005	-0.034	0.296	0.013	0.687
fit_3		0.254	<0.01	0.042	0.200	0.010	0.764	0.133	<0.01	-0.028	0.394	0.072	0.026
p	*HIV-*	0.719	<0.01	0.936	<0.01	-0.459	<0.01	-0.747	<0.01	-0.217	<0.01	0.498	<0.01
	HIV+/ART	0.257	<0.01	0.483	<0.01	0.070	0.032	-0.106	<0.01	-0.141	<0.01	0.164	<0.01
x_{TB}		0.427	<0.01	0.677	<0.01	0.010	0.758	-0.027	0.406	-0.147	<0.01	0.215	<0.01
ν	*HIV-*	-0.009	0.775	0.018	0.574	-0.021	0.520	0.009	0.775	0.024	0.465	0.041	0.211
	HIV+/ART	-0.111	<0.01	-0.257	<0.01	-0.140	<0.01	-0.258	<0.01	0.066	0.043	0.269	<0.01
τ	*HIV-*	0.032	0.330	0.157	<0.01	-0.015	0.645	-0.073	0.026	0.016	0.629	-0.014	0.673
	HIV+/ART	-0.025	0.443	-0.079	0.015	0.009	0.787	0.024	0.462	0.018	0.589	0.004	0.907
$\frac{1}{\kappa}$		-0.010	0.763	0.051	0.118	0.550	<0.01	0.787	<0.01	0.114	<0.01	0.187	<0.01
ω_1		-0.137	<0.01	-0.028	0.389	-0.108	<0.01	0.015	0.653	-0.003	0.921	-0.035	0.283
ω_2		-0.033	0.314	0.022	0.493	0.046	0.160	-0.178	<0.01	0.016	0.623	-0.006	0.846
$\frac{1}{\rho_1}$	*DS-TB*	-0.025	0.436	-0.010	0.748	0.017	0.608	-0.017	0.600	0.028	0.392	0.032	0.329
$\frac{1}{\rho_2}$	*MDR-TB*	0.002	0.948	0.015	0.641	0.016	0.621	-0.001	0.972	0.048	0.139	0.014	0.663
$\frac{1}{\rho_3}$	*XDR-TB*	-0.035	0.282	-0.042	0.193	-0.007	0.828	-0.019	0.556	0.016	0.622	-0.033	0.313
q_{11}	*DS-TB*	0.101	<0.01	0.154	<0.01	0.025	0.448	-0.027	0.414	-0.017	0.600	-0.035	0.278
q_{12} , q_{22}	*MDR-TB*	0.141	<0.01	0.011	0.727	-0.058	0.073	0.022	0.496	-0.062	0.055	0.006	0.851
q_{33}	*XDR-TB*	0.069	0.033	0.026	0.431	0.012	0.722	0.337	<0.01	-0.013	0.694	0.030	0.365
x_{HIV}		-0.104	<0.01	-0.118	<0.01	-0.067	0.040	-0.071	0.028	-0.789	<0.01	-0.105	<0.01
ϕ_{TB1}	*DS-TB*	0.183	<0.01	0.194	<0.01	-0.386	<0.01	-0.467	<0.01	-0.105	<0.01	0.055	0.093
$\phi_{TB2/3}$	*MDR- & XDR-TB*	-0.563	<0.01	0.084	0.010	-0.827	<0.01	-0.871	<0.01	0.014	0.659	-0.243	<0.01
d_{TB1}	*DS-TB*	0.041	0.211	-0.009	0.783	0.005	0.880	0.022	0.491	-0.016	0.633	0.032	0.321
$d_{TB2/3}$	*MDR- & XDR-TB*	0.002	0.959	-0.001	0.967	-0.025	0.434	0.018	0.588	0.001	0.985	0.002	0.959

d_{HIV}		-0.015	0.656	-0.040	0.217	0.013	0.690	0.021	0.515	0.032	0.320	0.052	0.111
$\frac{1}{\gamma}$	*TB-*	0.121	<0.01	0.121	<0.01	0.014	0.661	0.116	<0.01	0.694	<0.01	0.155	<0.01
	TB+	-0.040	0.219	0.028	0.397	-0.056	0.083	0.024	0.463	0.003	0.937	0.019	0.551
f	*HIV-*	0.097	<0.01	0.275	<0.01	-0.108	<0.01	-0.210	<0.01	-0.057	0.081	0.093	0.004
	HIV+/ART	0.341	<0.01	0.576	<0.01	-0.014	0.671	-0.285	<0.01	-0.118	<0.01	0.257	<0.01
ζ		-0.115	<0.01	-0.207	<0.01	-0.060	0.065	-0.065	0.045	0.009	0.787	-0.062	0.057
w		-0.018	0.591	0.071	0.029	-0.041	0.210	-0.038	0.239	0.000	0.991	-0.005	0.874
υ		-0.119	<0.01	-0.200	<0.01	-0.102	<0.01	-0.078	0.017	-0.012	0.710	0.001	0.979
β_{TB}		0.240	<0.01	0.525	<0.01	-0.138	<0.01	-0.280	<0.01	-0.073	0.024	0.117	<0.01
β_{HIV}		0.206	<0.01	0.286	<0.01	0.131	<0.01	0.013	0.700	-0.332	<0.01	0.445	<0.01
μ	*HIV-*	0.023	0.482	-0.039	0.227	-0.012	0.711	0.050	0.123	0.033	0.305	-0.023	0.481
	HIV+ or ART CD4 >350	-0.078	0.017	-0.163	<0.01	-0.012	0.717	0.190	<0.01	0.428	<0.01	-0.799	<0.01
	ART CD4 ≤350	0.054	0.095	0.032	0.332	0.054	0.098	0.089	<0.01	0.128	<0.01	-0.795	<0.01
	HIV+ CD4 ≤350	-0.602	<0.01	-0.693	<0.01	-0.441	<0.01	-0.395	<0.01	-0.906	<0.01	0.020	0.532
μ_{TB}	*HIV-*	-0.092	<0.01	-0.323	<0.01	-0.050	0.126	-0.026	0.430	0.010	0.765	0.023	0.487
	HIV+	-0.146	<0.01	-0.132	<0.01	-0.116	<0.01	-0.094	<0.01	-0.008	0.801	-0.046	0.158
	ART	0.139	<0.01	-0.146	<0.01	0.286	<0.01	0.529	<0.01	-0.006	0.862	0.007	0.822
δ		-0.432	<0.01	-0.548	<0.01	0.205	<0.01	0.299	<0.01	0.203	<0.01	-0.189	<0.01
η		-0.019	0.567	-0.023	0.474	-0.063	0.054	-0.049	0.136	-0.239	<0.01	-0.105	<0.01
vl		0.285	<0.01	0.363	<0.01	0.173	<0.01	0.123	<0.01	0.828	<0.01	0.443	<0.01
α		-0.241	<0.01	-0.263	<0.01	-0.085	<0.01	-0.016	0.620	0.344	<0.01	-0.433	<0.01
g		0.220	<0.01	0.453	<0.01	-0.001	0.969	-0.154	<0.01	0.036	0.269	0.253	<0.01
treat		0.490	<0.01	0.467	<0.01	0.492	<0.01	0.420	<0.01	0.004	0.905	0.056	0.088

S4 Table. Modeled Disease States.

Disease State	*Description*
TB	
S	Susceptible
P_S	Susceptible receiving IPT
L	Latent infection, slow
E	Latent infection, fast
P_L	Latent infection receiving IPT
A	Active infection
T	Active infection on effective TB treatment
F	Active infection in ineffective TB treatment
D	Active infection detected and awaiting culture/DST results
R	Recovered
P_R	Recovered receiving IPT
HIV	
S	Susceptible
I	Infected, CD4+ cell count > 350 cells/ml
E	Infected, CD4+ cell count ≤ 350 cells/ml
I_{ART}	Infected and on ART at CD4+ cell count > 350 cells/ml
E_{ART}	Infected and on ART at CD4+ cell count ≤ 350 cells/ml
